# Twist1 induces chromosomal instability (CIN) in colorectal cancer cells

**DOI:** 10.1093/hmg/ddaa076

**Published:** 2020-04-27

**Authors:** Maithilee Khot, Dyuthi Sreekumar, Sanika Jahagirdar, Apoorva Kulkarni, Kishore Hari, Elangoli Ebrahimkutty Faseela, Radhakrishnan Sabarinathan, Mohit Kumar Jolly, Kundan Sengupta

**Affiliations:** 1 B-216, Chromosome Biology Lab (CBL), Indian Institute of Science Education and Research (IISER), Dr Homi Bhabha Road, Pashan, Pune 411008, India; 2 Center for BioSystems Science and Engineering, Indian Institute of Science, Bengaluru 560012, India; 3 National Centre for Biological Sciences, Tata Institute of Fundamental Research, Bengaluru 560065, India

## Abstract

Twist1 is a basic helix-loop-helix transcription factor, essential during early development in mammals. While Twist1 induces epithelial-to-mesenchymal transition (EMT), here we show that Twist1 overexpression enhances nuclear and mitotic aberrations. This is accompanied by an increase in whole chromosomal copy number gains and losses, underscoring the role of Twist1 in inducing chromosomal instability (CIN) in colorectal cancer cells. Array comparative genomic hybridization (array CGH) analysis further shows sub-chromosomal deletions, consistent with an increased frequency of DNA double strand breaks (DSBs). Remarkably, Twist1 overexpression downmodulates key cell cycle checkpoint factors—Bub1, BubR1, Mad1 and Mad2—that regulate CIN. Mathematical simulations using the RACIPE tool show a negative correlation of Twist1 with E-cadherin and BubR1. Data analyses of gene expression profiles of patient samples from The Cancer Genome Atlas (TCGA) reveal a positive correlation between Twist1 and mesenchymal genes across cancers, whereas the correlation of TWIST1 with CIN and DSB genes is cancer subtype-specific. Taken together, these studies highlight the mechanistic involvement of Twist1 in the deregulation of factors that maintain genome stability during EMT in colorectal cancer cells. Twist1 overexpression enhances genome instability in the context of EMT that further contributes to cellular heterogeneity. In addition, these studies imply that Twist1 downmodulates nuclear lamins that further alter spatiotemporal organization of the cancer genome and epigenome. Notwithstanding their genetic background, colorectal cancer cells nevertheless maintain their overall ploidy, while the downstream effects of Twist1 enhance CIN and DNA damage enriching for sub-populations of aggressive cancer cells.

## Introduction

Twist1 is a basic helix-loop-helix (bHLH) transcription factor that is essential for normal vertebrate development, but is overexpressed in cancers of the breast, prostate and stomach, including melanomas, gliomas and osteosarcomas (1,2). Increase in Twist1 levels is implicated in dissemination of tumorigenic cells and chemoresistance (3). Twist1 is a master regulator of epithelial-to-mesenchymal transition (EMT) (4) and promotes stemness (5)—a characteristic feature of EMT (6–8). Twist1 binds to the promoter of the E-cadherin gene (that encodes for a cell adhesion protein) and suppresses its expression (9). Decrease in E-cadherin levels reduces the cobblestone morphology of epithelial cells, also facilitating their dissemination (10). Consistently, a subpopulation of breast, colorectal, prostate and lung carcinomas shows Twist1 expression, typically at the invasive edge of cells (11). As Twist1 drives tumor progression, its contribution to EMT is extensively studied across cancers (4). However, the impact of Twist1 overexpression on chromosomal stability in the context of EMT in cancer cells remains unclear.

Twist1 overexpression induces chromosomal instability (CIN) in cancers of the breast (12). Spectral karyotyping (SKY) analyses of metaphases derived from Twist1 overexpressing MCF-7 (breast cancer cell line) showed an increase in chromosomal aberrations such as aneuploidy and translocations (13). Consistent with this observation, the stroma of colorectal tumors shows a positive correlation between Twist1 positive cells and CIN (14). However, the underlying mechanisms of Twist1-induced CIN remain elusive.

Another interesting vignette in our understanding of the mechanistic basis of CIN also has its origins in the maintenance of the morphology and function of the nucleus by the type V intermediate filament proteins—Lamins A/C, B1 and B2 that are localized at the inner nuclear envelope (15,16). Mutations or loss of lamins strikingly alter nuclear shapes resulting in aberrant nuclei, nuclear blebs and micronuclei, which are precursors of CIN (17). Lamin loss also impacts the cellular transcriptome (18). Interestingly, Lamin B2 knockdown shows chromosomal gains in the otherwise diploid colorectal cancer cells (DLD1) (19). Furthermore, Lamin B2 depletion shows chromosomal imbalances in colorectal cancer cells and associates with the spindle machinery, further suggesting the role of lamins in chromosome segregation in mitotic cells (20). However, the mechanisms underlying lamin functions in chromosomal stability in cancer cells are unclear.

Colorectal cancers show microsatellite instability (MSI), characterized by the insertion of repetitive nucleotide stretches, typically corrected by proteins of the mismatch repair system (MMR) such as MSH2, MSH6, MLH1 and PMS2 (21). Colorectal cancers that are mismatch repair-deficient (MMR−) show high microsatellite instability (MSI+), while mismatch repair-proficient (MMR+) colorectal cancers do not show microsatellite instability, but show elevated levels of CIN (21).

The cell cycle checkpoint and tumor suppressor protein p53 is essential for the maintenance of chromosomal stability across cancers (22,23). Furthermore, the status of p53 is potentially an important determinant of CIN, since cells with mutant p53 are associated with CIN, while cells with wild type p53 show significantly reduced CIN in cancer cells (22,24). Evidence of CIN induction exists even in the presence of wild type p53, suggesting alternate pathways of CIN induction in cancer cells (13,25). Reduction in p53 levels also enhances the susceptibility of cells to DNA damage, as ascertained by an increase in γH2AX foci (26).

With the wealth of patient data available from The Cancer Genome Atlas (TCGA)—various molecular correlates that range from mutations, copy number alterations, and expression status among others—can be attributed to target genes in specific cancer subtypes (27). Furthermore, mathematical modelling and simulations have the power to compute and predict the potential outcome of novel molecular interactions and their pathways involved in actively promoting cancers. It is therefore beyond any doubt that an interdisciplinary approach of studying theoretical and experimental paradigms is essential for cancer intervention.

Here, we show that Twist1 overexpression induces EMT to varying extents in the two colorectal cancer cell lines. Furthermore, Twist1 overexpression significantly increases nuclear and mitotic aberrations, accompanied by an increase in CIN. In addition, Twist1 induces sub-chromosomal deletions, consistent with an increase in DNA double strand breaks, as revealed by an increase in γH2AX foci. Twist1 overexpression showed a significant decrease in the levels of Spindle Assembly Checkpoint (SAC) proteins such as Bub1/R1, Mad1/2 and Aurora B Kinase, and the p53 oncoprotein, underscoring their collective role in regulating chromosomal stability in colorectal cancer cells. This was also corroborated by mathematical simulations, which showed a negative correlation between the levels of Twist1 and BubR1. Taken together, our studies suggest an overarching role of Twist1 in modulating chromosomal stability in colorectal cancer cells.

## Results

### Twist1 overexpression shows differential induction of EMT in colorectal cancer cells

**Figure 1:**
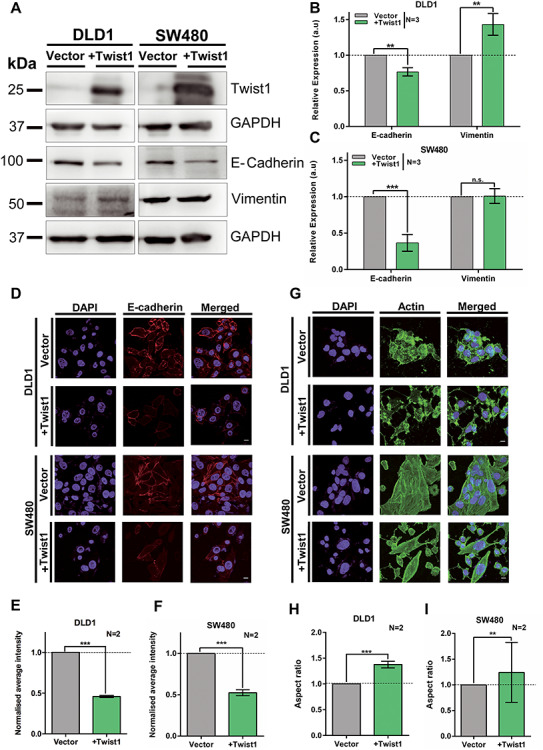
Differential induction of EMT upon Twist1 overexpression in colorectal cancer cells. (**A**) Representative immunoblot showing Twist1 overexpression with a concomitant decrease in E-cadherin and marginal increase in Vimentin levels in DLD1 and SW480 cell lines. (**B** and **C**) Quantification of band intensities of E-cadherin and Vimentin protein levels in DLD1 and SW480 cell lines upon Twist1 overexpression. Data from three independent biological replicates normalized to GAPDH (unpaired *t*-test, *N* = 3, mean ± SD, ^*^P < 0.05, ^**^P < 0.01, ^***^P < 0.001 and ^****^P < 0.0001). (**D**) Representative mid-optical sections from confocal z-stacks of DLD1 and SW480 cells immunostained for E-cadherin, scale bar ~10 μm. (**E** and **F**) Normalized fluorescence intensity of E-cadherin for vector and Twist1 overexpressing cells. Data from two independent biological replicates for DLD1 and SW480, respectively, (Mann–Whitney test, *N* = 2, *n* > 60, mean ± SD, ^*^P < 0.05, ^**^P < 0.01, ^***^P < 0.001 and ^****^P < 0.0001). (**G**) Immunostaining for actin showing an elongated and spindle-shaped morphology upon Twist1 overexpression, scale bar ~10 μm. (**H** and **I**) Quantification of aspect ratio of cells (Mann–Whitney test, *N* = 2, *n* > 40, mean ± SD, ^*^P < 0.05, ^**^P < 0.01, ^***^P < 0.001 and ^****^P < 0.0001). *N*: number of independent biological replicates, *n*: number of cells.

The role of Twist1 is well established in EMT during early development and cancer progression. Transient overexpression was preferred over stable expression as a model to mimic the heterogeneous upsurge in the levels of Twist1 during cancer progression (28). We therefore studied the effect of transiently overexpressing Twist1 in two independent colorectal cancer cell lines—(i) DLD1—a near diploid, mismatch repair-deficient cell line and (ii) SW480—aneuploid, mismatch repair proficient cell line. We independently transfected these two cell lines with Twist1 and examined Twist1 protein levels by immunoblotting, which showed a significant increase in both cell lines (Fig. 1A). Consistent with previous results, Twist1 overexpression showed a significant decrease in the levels of the epithelial marker—E-cadherin (~30%), and an increase in the expression levels of the bonafide mesenchymal marker—Vimentin (~43%) in DLD1 cells (Fig. 1B). In contrast, the hyperdiploid colorectal cancer cell line SW480, showed a marked decrease (~64%) in E-cadherin levels, but only a marginal increase in Vimentin levels (Fig. 1C). We also examined the status of EMT induction at the single cell level by performing immunofluorescence staining. E-cadherin levels showed a significant decrease in both DLD1 (~50%) and SW480 cells (~45%) (Fig. 1D–F), underscoring that the decrease in epithelial mark(s) is an important event in EMT. Furthermore, these cells showed an increase in aspect ratio (DLD1 ~ 30%, SW480 ~ 24%)—a characteristic feature of cell elongation as quantified from phalloidin labelled cells (Fig. 1G–I). In summary, colorectal cancer cells exhibit EMT to varying extents upon Twist1 overexpression.

### Nuclear and mitotic aberrations are enhanced in colorectal cancer cells upon Twist1 overexpression

**Figure 2:**
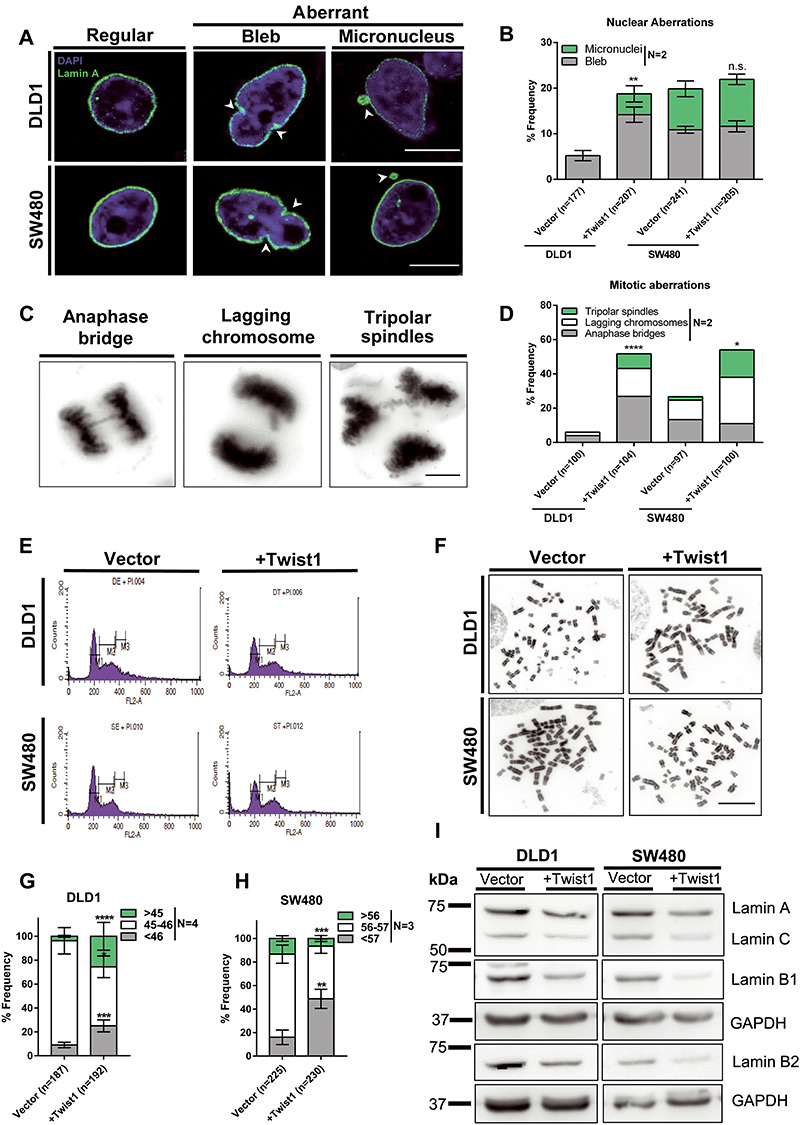
Twist1 overexpression enhances nuclear and mitotic aberrations in colorectal cancer cells. (**A**) Representative confocal images of nuclei upon Twist1 overexpression in DLD1 and SW480 cells immunostained with Lamin A showing nuclear blebs and micronuclei, scale bar ~10 μm. (**B**) Quantification of number of cells showing aberrant nuclei upon Twist1 overexpression. Data quantified from two independent biological replicates (Chi-square test, *N* = 2, mean with range, ^*^P < 0.05, ^**^P < 0.01, ^***^P < 0.001 and ^****^P < 0.0001). (**C**) Representative images of mitotic aberrations showing anaphase bridges, lagging chromosomes and triploar spindles, scale bar ~10 μm. (**D**) Quantification of the number of mitotic aberrations upon Twist1 overexpression. Data quantified from two independent biological replicates (Chi-square test, *N* = 2, mean, ^*^P < 0.05, ^**^P < 0.01, ^***^P < 0.001 and ^****^P < 0.0001). (**E**) Representative flow cytometry profiles for ploidy analysis of vector and Twist1 overexpressing cells (*N* = 2). (**F**) Representative images of metaphase chromosome spreads derived from DLD1 and SW480 cell lines upon Twist1 overexpression, scale bar ~10 μm. (**G** and **H**) Quantification of whole chromosomal gains and losses for DLD1 and SW480 cells, respectively (data quantified from *n* > 180 independent metaphase spreads collected from *N* = 3 independent biological replicates, *Z*-test of proportions, mean ± SEM, ^*^P < 0.05, ^**^P < 0.01, ^***^P < 0.001 and ^****^P < 0.0001). (**I**) Representative immunoblot showing downregulation of lamin levels upon Twist1 overexpression (*N* = 2).

Aberrant nuclear morphologies such as nuclear blebs and micronuclei are enhanced in cancers and characterize cancer progression (29). The frequency of such aberrant nuclear morphologies are diagnostic features, quantified in histopathological analyses of tissue biopsy samples (30). In addition to inducing EMT, Twist1 is also an oncoprotein (3). Here we overexpressed Twist1 and determined the frequency of nuclear blebs and micronuclei in colorectal cancer cells (Fig. 2A and B and Supplementary Material, Table S1). While there was an increase (~5%) in the frequency of micronuclei and nuclear blebs (~9%) in DLD1 cells, SW480 cells hardly showed an increase in these aberrations (Fig. 2B). Since aberrant nuclei are also precursors of CIN, we determined the extent of mitotic aberrations upon Twist1 overexpression (17,31). Interestingly, near diploid DLD1 cells showed a significant increase in the extent of mitotic aberrations than SW480 cells (Fig. 2C and D). Furthermore, DLD1 cells showed an increase in anaphase bridges (~23%), lagging chromosomes (~14%) and tripolar spindles (~9%), while SW480 cells showed a decrease (~3%) in anaphase bridges, accompanied by an increase in lagging chromosomes (~16%) and tripolar spindles (~14%), respectively (Fig. 2D and Supplementary Material, Table S2).

Having found a significant increase in mitotic aberrations associated with Twist1 overexpression, we asked if Twist1 induces CIN in colorectal cancer cells. We first analyzed the ploidy of cells upon Twist1 overexpression by flow cytometry. Neither DLD1 nor SW480 cells showed changes in their overall ploidy, upon Twist1 overexpression for ~72 h (Fig. 2E and Supplementary Material, Fig. S1E and F).

However, we detected a significant increase in the number of cells showing whole chromosomal gains (~23%) and losses (~16%), upon Twist1 overexpression in DLD1 cells (Fig. 2F and G). In contrast, there was a significant increase in the number of SW480 cells, showing whole chromosomal losses (~32%), but a decrease in cells with whole chromosome gains (~7%) upon Twist1 overexpression (Fig. 2F and H and Supplementary Material, Fig. S2C and D, Table S3).

We asked if Twist1 overexpression also induces CIN in another near diploid colorectal cancer cell line—HCT116 (i) wild type for p53 and (ii) shows microsatellite instability (MSI+). Remarkably, HCT116 cells did not show any change in their modal chromosome numbers of 42–43, upon Twist1 overexpression (Supplementary Material, Fig. S3). This is consistent with an overarching role for wild type p53 protein in the maintenance of chromosomal stability in colorectal cancer cells.

Nuclear lamins (Lamin A/C, B1 and B2) localized at the inner nuclear envelope maintain nuclear structure and function (16). Lamins also modulate chromosomal stability in colorectal cancer cells (19,20). While immunoblotting assays show comparable levels of all three subtypes of nuclear lamins in DLD1 cells, in contrast, SW480 cells show reduced levels of endogenous B-type lamins (Fig. 2I). Interestingly, lamin levels decreased in both cell lines, with B-type lamins showing a further decrease in SW480 as compared to DLD1 cells upon Twist1 overexpression (Supplementary Material, Fig. S2E and F). These results suggest that Twist1 overexpression decreases B-type lamins levels, consistent with an increase in aberrant nuclear shapes and CIN upon loss of B-type lamins in colorectal cancer cells.

In summary, CIN is induced in a differential manner in the two colorectal cancer cell lines, upon Twist1 overexpression, since DLD1 cells exhibit both whole chromosomal gains and losses, while SW480 cells predominantly show whole chromosomal losses (Fig. 2F–H). Taken together, Twist1 overexpression induces and enhances levels of nuclear aberrations and CIN in colorectal cancer cells.

### Genome-wide increase in sub-chromosomal deletions upon Twist1 overexpression

**Figure 3:**
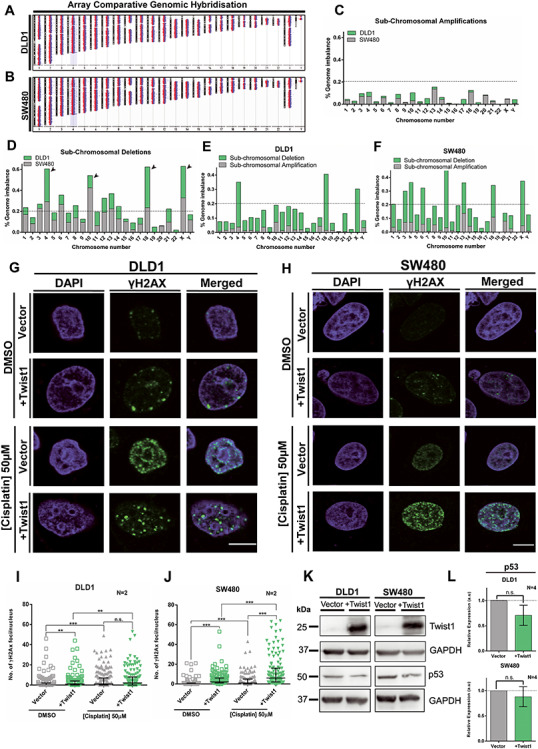
Twist1 overexpression induces sub-chromosomal deletions. (**A** and **B**) Chromosomal ideograms showing sub-chromosomal deletions and amplifications derived from array comparative genomic hybridisation (array CGH) for Twist1 overexpressing DLD1 and SW480 cells, respectively. (**C**) Sub-chromosomal amplifications quantified for each chromosome normalized to its total DNA content for DLD1 and SW480 cells. (**D**) Sub-chromosomal deletions quantified for each chromosome normalized to its total DNA content for DLD1 and SW480 cells. (**E**) Sub-chromosomal amplifications and deletions quantified for each chromosome normalized to its total DNA content for DLD1. (**F**) Sub-chromosomal amplifications and deletions quantified for each chromosome normalized to its total DNA content for SW480. The array CGH was from two independent biological replicates (*N* = 2, mean). (**G**) Representative mid-optical sections of DLD1 cells immunostained for γH2AX foci upon Twist1 overexpression upon DNA damage induction upon cisplatin treatment, vehicle control (DMSO), scale bar ~10 μm. (**H**) Representative mid-optical sections of SW480 cells immunostained for γH2AX foci upon Twist1 overexpression upon DNA damage induced upon cisplatin treatment, vehicle control (DMSO), scale bar ~10 μm. (**I** and **J**) Quantification of γH2AX foci in (I) DLD1 and (J) SW480 cells, respectively (Mann–Whitney test, *N* = 2, *n* > 130, Median-IQR, ^*^P < 0.05, ^**^P < 0.01, ^***^P < 0.001 and ^****^P < 0.0001, *n*: number of nuclei). (**K**) A representative immunoblot showing p53 levels upon Twist1 overexpression in DLD1 and SW480 cells. (**L**) Quantification of p53 protein levels from band intensities normalized to GAPDH (unpaired *t*-test, *N* = 4, mean ± SD, ^*^P < 0.05, ^**^P < 0.01, ^***^P < 0.001 and ^****^P < 0.0001), *N*: number of independent biological replicates.

Our studies unravel a positive correlation between Twist1 overexpression and CIN in colorectal cancer cells. We therefore performed array-comparative genomic hybridization (array CGH) as an independent approach to determine the extent of amplifications and deletions at the sub-chromosomal level across the genome (Fig. 3A and B). Cells were subjected to EMT induction upon Twist1 overexpression, followed by array CGH analyses, while cells transfected with the corresponding empty vector served as reference. Analysis of array CGH data revealed sub-chromosomal amplifications and deletions across the genome (Fig. 3C–F). Sub-chromosomal deletions were more prevalent upon Twist1 overexpression (Fig. 3C and D). Surprisingly, human Chr.4, Chr.10, Chr.18 and Chr.X showed a significantly greater extent of sub-chromosomal deletions in DLD1 cells as compared to other chromosomes (Fig. 3E). SW480 cells on the other hand showed a larger repertoire of sub-chromosomal deletions that predominantly map to human Chr.3, Chr.4, Chr.6, Chr.10, Chr.13, Chr.18 and Chr.X (Fig. 3F). Deletions occurred primarily in chromosomes 4, 10, 18 and *X* consistently in both cell lines upon Twist1 overexpression (Fig. 3E and F). Of note, the extent of sub-chromosomal deletions was considerably elevated in SW480 than DLD1 cells. An independent array CGH analyses performed on colorectal cancer patient tumors identified copy number aberrations and exhibited sub-chromosomal deletions in human chromosomes 4, 8, and 18, respectively (32). In summary, array CGH analyses revealed a significant increase in the frequency of sub-chromosomal deletions upon Twist1 overexpression—an additional contributor of CIN (33).

### Twist1 overexpression induces DNA damage and downregulates p53

A noteworthy finding from genome wide array CGH analyses was the striking increase in sub-chromosomal deletions across the genome upon Twist1 overexpression. Since chromosomal aberrations such as deletions are consequences of DSB formation (34,35), we sought to examine whether Twist1 overexpression induces DNA DSBs in colorectal cancer cells (Fig. 3G and H). We monitored the number of γH2AX foci in single cells as a marker of DNA Double Strand Breaks (DSBs) upon Twist1 overexpression and upon cisplatin treatment (50 μm), by immunofluorescence assays. Interestingly, Twist1 overexpression in DLD1 cells showed a significant increase in the number of γH2AX foci in the interphase nucleus (Fig. 3I). However, cisplatin treatment in the background of Twist1 overexpression did not alter the number of DNA damage foci (Fig. 3I). In contrast, Twist1 overexpression in SW480 cells showed a significant increase in γH2AX foci independently and in the presence of cisplatin (Fig. 3J). Taken together, this suggests that Twist1 overexpression induces and enhances DNA double strand breaks in colorectal cancer cells (Fig. 3G–J).

Since p53 is a master regulator of genome integrity in mammalian cells (36,37), we determined the effect of Twist1 overexpression on the levels of p53. Interestingly, Twist1 overexpression showed a decrease in p53 levels in DLD1 and a marginal decline in SW480 cells (Fig. 3K and L). Taken together, this suggests that the decrease in p53 levels potentially predisposes cells to elevated levels of DNA damage in cancer cells.

### Twist1 overexpression downregulates checkpoint proteins

**Figure 4:**
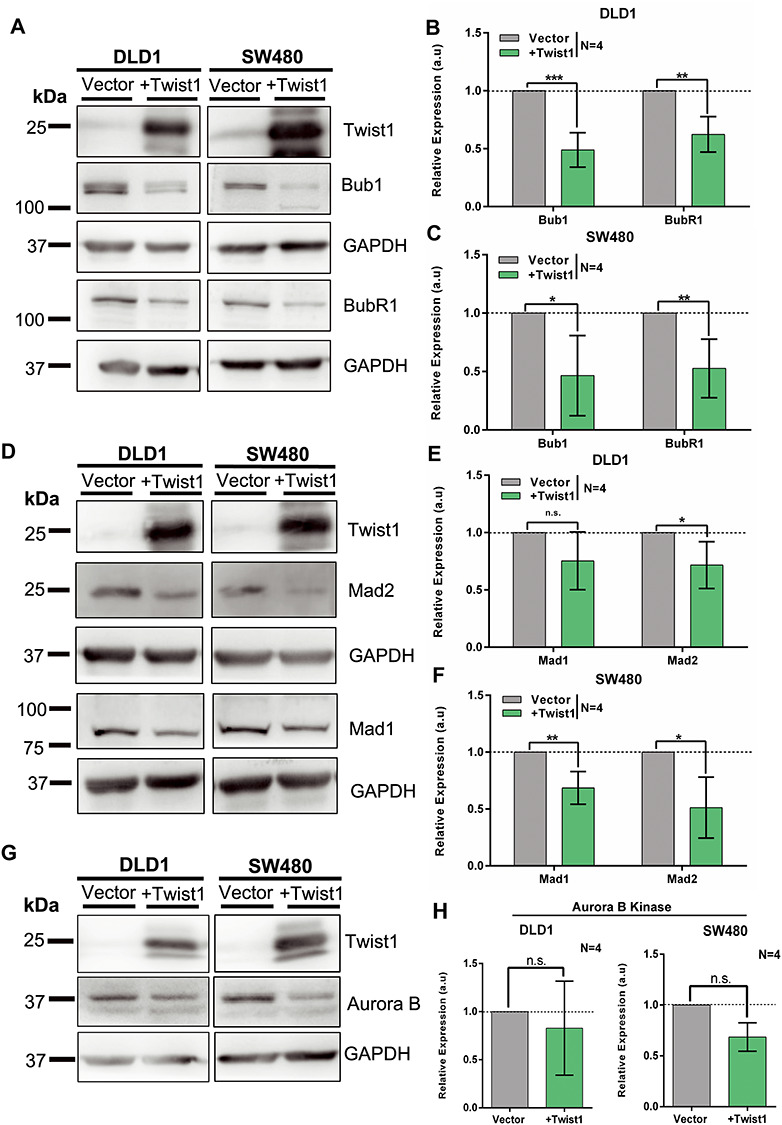
Twist1 overexpression shows a decrease in checkpoint proteins. (**A**) A representative immunoblot showing Twist1 overexpression, accompanied by a significant decrease in Bub1 and BubR1 levels in DLD1 and SW480 cells. (**B** and **C**) Quantification of Bub1 and BubR1 protein levels from (B) DLD1 and (C) SW480 cells, calculated from band intensities normalized to GAPDH (unpaired *t*-test, *N* = 4, mean ± SD, ^*^P < 0.05, ^**^P < 0.01, ^***^P < 0.001 and ^****^P < 0.0001). (**D**) A representative immunoblot showing Twist1 overexpression, accompanied by decrease in mitotic checkpoint proteins Mad1 and Mad2 in DLD1 and SW480 cells, respectively. (**E** and **F**) Quantification of Mad1 and Mad2 protein levels from (E) DLD1 and (F) SW480 cells, calculated from band intensities normalized to GAPDH (unpaired *t*-test, *N* = 4, mean ± SD, ^*^P < 0.05, ^**^P < 0.01, ^***^P < 0.001 and ^****^P < 0.0001). (**G**) A representative immunoblot showing Twist1 overexpression and decrease in Aurora B Kinase levels, in DLD1 and SW480 cells. (**H**) Quantification of Aurora B Kinase protein levels (unpaired *t*-test, normalized to loading control GAPDH, *N* = 4, mean ± SD, ^*^P < 0.05, ^**^P < 0.01, ^***^P < 0.001 and ^****^P < 0.0001).

We sought to address the underlying mechanisms leading to CIN upon Twist1 overexpression. As we detected a significant increase in mitotic defects and whole chromosomal aberrations, we monitored the levels of Spindle Assembly Checkpoint (SAC) proteins namely, Bub1, BubR1, Mad1, Mad2 and Aurora B Kinase (38–42). We overexpressed Twist1 independently in the two colorectal cancer cell lines and performed immunoblotting on whole cell extracts derived from these cells. Remarkably, the levels of the Bub1 and BubR1 proteins of the SAC showed a significant and comparable decrease in both colorectal cancer cell lines upon Twist1 overexpression (Fig. 4A–C). Mad1 and Mad2—components of the mitotic checkpoint complex—also showed a decrease upon Twist1 overexpression (Fig. 4D–F). In addition, Aurora B Kinase—a part of the chromosome passenger complex—also showed a decrease in protein levels (Fig. 4G and H). CIN is a consistent feature associated with the deregulation of Bub1/BubR1 levels (43–45), and decrease in their levels further affects the levels of downstream proteins such as Mad1/2 (38,46). In summary, Twist1 overexpression shows a decrease in the levels of CIN regulators, which further underscores the contribution of Twist1 to CIN in colorectal cancer cells.

We asked if the decreased levels of checkpoint proteins upon Twist1 overexpression were also elicited at the transcript level. We therefore performed RT-PCR analyses of checkpoint genes upon Twist1 overexpression. Interestingly, BUB1, BUBR1, MAD2L1 and AURKB showed a significant decrease in their transcript levels in both DLD1 and SW480 cell lines (Supplementary Material, Fig. S4B, C, E and F). In contrast, MAD1L1 showed a differential response as it was downregulated in DLD1 but significantly upregulated in SW480 cells upon Twist1 overexpression (Supplementary Material, Fig. S4D). In summary, Twist1 overexpression represses checkpoint genes at the transcriptional level, otherwise required for the maintenance of chromosomal stability of colorectal cancer cells.

### Twist1 impinges on CIN regulation

To address the potential crosstalk between Twist1 and the regulators of chromosomal stability, we performed network analyses of (i) generic protein–protein interactions (47), (ii) transcription factor (TF)–gene interactions (48) of Twist1 and factors associated with EMT, CIN and DNA damage using NetworkAnalyst—a visual data analytics platform (Supplementary Material, Fig. S5A and B). From protein–protein interaction network analyses, p53 emerges as a major hub through which Twist1 regulates CIN factors, since Twist1 affects the DNA-binding activity of p53, thereby impairing its function (11). In addition, p53 directly interacts with Aurora B Kinase and Bub1, while interacting with Mad2 via FZR1 (Supplementary Material, Fig. S5A). Bub1 also interacts with Mad1 and BubR1 (39,46). Twist1 shows a potentially indirect interaction with Lamins via p53 and SUMO1—a post-translation protein modifier, and CDK1—a cell cycle regulator (Supplementary Material, Fig. S5A).

ENCODE ChIP-Seq data for transcription factor (TF) enrichment on target genes (Supplementary Material, Fig. S5B) show that Twist1 may modulate TP53 activity via the histone modifier SUZ12- part of the polycomb repressive complex 2 (PRC2). Furthermore, Twist1 indirectly modulates Aurora B Kinase activity via SP3—a transcriptional repressor/activator (49). Additionally, Twist1 modulates CDH1 (E-cadherin) via EZH2 (component of the PRC2) (50). Twist1 differentially regulates lamins (LMNA, LMNB2) through the transcriptional repressor CTBP2 (51). Transcription factor—RFXANK is enriched on LMNB2 and MAD2L1 genes. Also, NR4A1—a nuclear transcription factor—emerges as a modulator of VIM (Vimentin) and BUB1. In summary, while Twist1 functions as a transcription factor, its protein–protein interaction analyses highlight p53 as a central node, further suggestive of the role of Twist1 in modulating CIN via p53.

### A simulation-based approach shows negative correlation between Twist1 and E-cadherin and BubR1 levels

**Figure 5:**
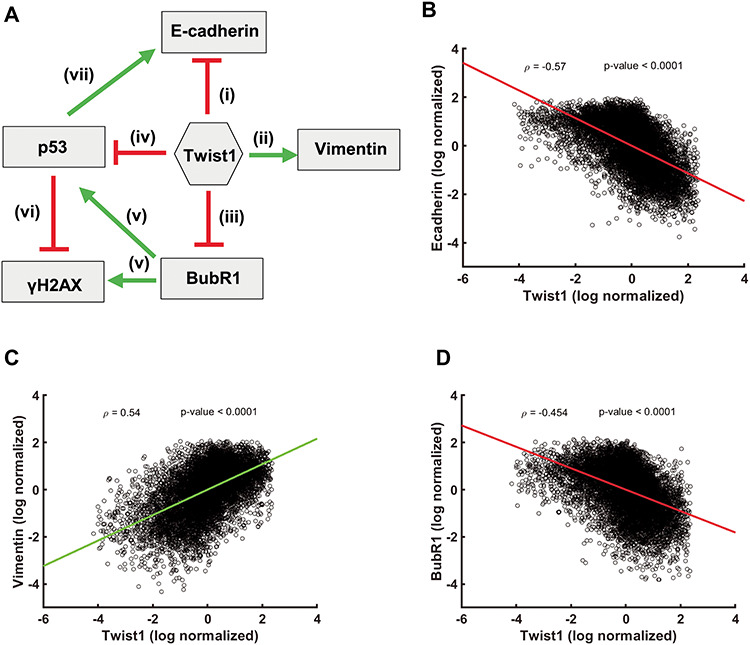
Correlation between levels of Twist1, EMT and CIN factors. (**A**) Network depicting the interactions among Twist1, EMT and CIN genes. Correlation plots of the log normalized gene expression values of (**B**) Twist1 and E-cadherin (**C**) Twist1 and Vimentin (**D**) Twist1 and BubR1, ρ = Pearson correlation coefficient, *P*-values show the significance of Pearson correlation.

We next constructed a regulatory network by integrating our experimental data with known interconnections among Twist1, E-cadherin, Vimentin, BubR1, γH2AX and p53 (Fig. 5A). (i) Twist1 overexpression downregulates E-cadherin by binding to its promoter (9) (ii) Twist1 overexpression upregulates Vimentin and is mediated by CUL2 (52) (iii) Twist1 overexpression in colorectal cancer cells downregulates BubR1 (Fig. 4A–C) (iv) Twist1 downregulates p53 levels (11) (v) BubR1 levels positively correlate with levels of p53 and γH2AX in response to DNA damage (53) (vi) p53 is required for repair of DNA damage and shows a negative correlation with γH2AX levels (26). We therefore sought to identify the robust dynamic features emerging from these interconnections. We simulated the network using the RACIPE tool (54) that models a given network using a system of ordinary differential equations (ODE). Each equation in the system represents the dynamics of a node in the network. The ODEs are then simulated for multiple parameter sets chosen randomly from a pre-defined, biologically relevant parameter space. This way, the tool allows us to capture the dynamics of the network while recapitulating the omnipresent cell-to-cell variability. The output of these simulations is the steady state values of each node, i.e. gene expression levels (Fig. 5B–D).

Across the sampled parameter sets, we observed a significant negative correlation for Twist1- E-cadherin (Fig. 5B) and positive correlation for Twist1-Vimentin (Fig. 5C). This suggests that although the extent of EMT induction via Twist1 is heterogeneous across single cells, an ensemble behavior shows robust induction of EMT by Twist1 by altering the levels of E-cadherin and Vimentin. Notwithstanding the intrinsic heterogeneity across cells, a systems biology approach corroborates our experimental data, which shows that Twist1 and BubR1 expression levels are negatively correlated (Fig. 5D).

### Twist1 overexpression positively correlates with EMT and CIN: The Cancer Genome Atlas (TCGA) analyses

We sought to ask if the expression levels of Twist1 correlate with levels of (i) EMT-associated genes, (ii) CIN genes, (iii) DNA double-strand break (DSB) genes and (iv) tumor mutation burden and copy number alterations (CNAs).

**Figure 6:**
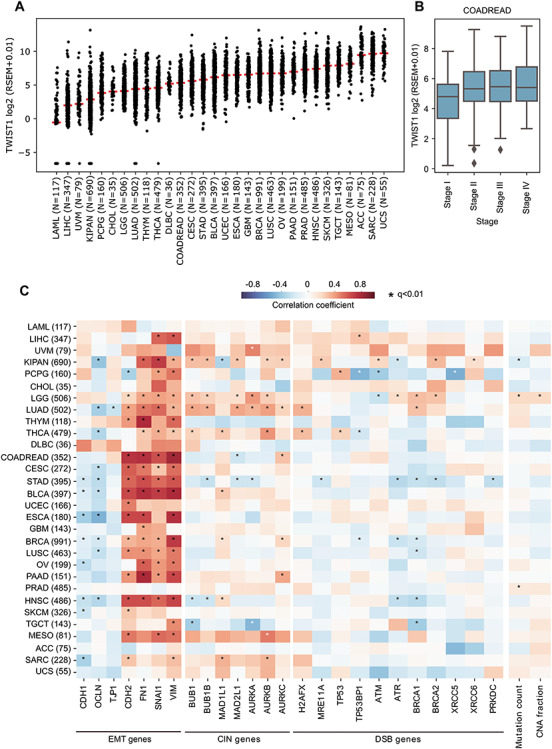
Correlation between gene expression levels of TWIST1, EMT and CIN across human cancers. (**A**) TWIST1 gene expression (log2 RSEM + 0.01) level within and across cancers of TCGA. Each dot represents a tumor sample and the horizontal red bar indicates the median expression value within that cancer cohort. The cancer type abbreviations are shown below. (**B**) TWIST1 expression in colorectal cancers (COADREAD) stratified by tumor stages. (**C**) Heatmap representing the correlation coefficient (and its significance) between TWIST1 expression and (i) EMT, (ii) CIN, (iii) DSB gene expression, (iv) mutation count and (v) copy number alteration (CNA) fraction for all 30 distinct cancers, computed using an iteratively reweighted least-squares approach. Color coding indicates the correlation coefficient, ranging from −1 to +1, where −1 being strong negative correlation (dark blue), 0 for no correlation (white) and +1 strong positive correlation (dark red). Significant correlations (*q* < 0.01) are marked with an asterisk (^*^). Cancer type abbreviations: ACC: adrenocortical carcinoma, BLCA: bladder urothelial carcinoma, BRCA: breast invasive carcinoma, CESC: cervical squamous cell carcinoma and endocervical adenocarcinoma, CHOL: cholangiocarcinoma, COADREAD: colorectal adenocarcinoma, DLBC: diffuse large B-cell lymphoma, ESCA: esophageal carcinoma, GBM: glioblastoma multiforme, HNSC: head and neck squamous cell carcinoma, KIPAN: pan kidney carcinomas, LAML: acute myeloid leukemia, LIHC: liver hepatocellular carcinoma, LGG: brain lower grade glioma, LUAD: lung adenocarcinoma, LUSC: lung squamous cell carcinoma, MESO: mesothelioma, OV: ovarian serous cystadenocarcinoma, PAAD: pancreatic adenocarcinoma, PCPG: pheochromocytoma and paraganglioma, PRAD: prostate adenocarcinoma, SARC: sarcoma, SKCM: skin cutaneous melanoma, STAD: stomach adenocarcinoma, TGCT: testicular germ cell tumors, THCA: thyroid carcinoma, THYM: thymoma, UCS: uterine carcinosarcoma, UCEC: uterine corpus endometrial carcinoma, UVM: uveal melanoma.

We analyzed the gene expression data and somatic mutations of 30 distinct cancer types from The Cancer Genome Atlas (TCGA). This shows that Twist1 expression is evident in many primary tumors, and that their level varies within and between cancer types, likely due to the cell-of-origin and tumor stage (Fig. 6A). For example, liver hepatocellular carcinoma (LIHC) and kidney cancers (KIPAN), which originate from epithelial cells, showed the least Twist1 expression, whereas sarcoma (SARC) and uterine carcinosarcoma (UCS) that originate from mesenchymal cells showed higher expression, as previously shown at the level of EMT gene signatures (55). Colorectal cancers (COADREAD) also showed a high expression of Twist1, especially in the late stage tumors (Fig. 6B). Furthermore, Twist1 expression positively correlates with EMT genes (CDH1, OCLN, TJP1, CDH2, FN1, SNAI1 and VIM) in various cancers (Fig. 6C). With CIN genes, we observed a significant positive correlation in certain tumor types, which include kidney cancers, lower-grade glioma (LGG) and lung adenocarcinoma (LUAD); whereas, in stomach adenocarcinoma (STAD) a significant negative correlation was observed between Twist1 and CIN genes. However, consistent with experimental data (Fig. 4D), colorectal cancers showed a moderate negative correlation between Twist1 and MAD2L1—a mitotic spindle assembly checkpoint protein. AURKC expression showed a marginal increase with Twist1 expression (Fig. 6C). However, we found a significant correlation with DSB genes in only a few tumor types (e.g. STAD showed a strong negative correlation similar to CIN genes).

We also examined if a correlation exists between Twist1 expression and CIN for which we compared Twist1 expression with tumor mutation burden and copy number alterations (CNAs). The total number of somatic point mutations and the fraction of genome with amplifications or deletions were considered as tumor mutation burden and CNA events, respectively. The cancer types LGG (Brain), KIPAN (Pan Kidney) and PRAD (prostate) showed a significant positive correlation with Twist1 expression for somatic mutation and for CNA events in LGG (Fig. 6C). In particular, LGG showed a significant positive correlation between TWIST1 with (i) EMT and genomic instability markers such as CIN and DSB gene expression and (ii) mutation burden and CNA event. Taken together, these results suggest that Twist1 expression is highly correlated with EMT in various cancers. However, the correlation between TWIST1 with CIN and DSB genes is cancer subtype-specific.

## Discussion

Twist1 is essential for the induction of EMT during the normal process of gastrulation during early development (4,56). However, Twist1 is overexpressed across cancers with a well-established role in metastasis (57). Here we show that Twist1 induces chromosomal and genomic instability in colorectal cancer cells (Fig. 2F–H). Twist1-induced CIN is characterized by both losses and gains of chromosomes in the near diploid DLD1 (CIN-) colorectal cancer cells, while the aneuploid SW480 (CIN+) colorectal cancer cells show chromosomal losses. Interestingly, a high-resolution approach of array comparative genomic hybridisation (array CGH) in addition to chromosomal imbalances also reveals extensive deletions at the sub-chromosomal level (Fig. 3A–F). Consistent with our results, Twist1 overexpression also induces CIN in MCF7 breast cancer cells. Spectral karyotyping (SKY) analyses show tetrasomy (~4 copies) of most chromosomes except human Chr.2, 3, 12, 18 and 21 upon Twist1 overexpression (13).

Twist1 overexpression shows a significant increase in nuclear aberrations such as nuclear blebs and micronuclei (Fig. 2A and B), consistent with decreased levels of nuclear lamins A/C and B2 (Fig. 2I). In addition, decrease in B-type lamins also induces CIN in colorectal cancer cell lines (19,20), suggestive of the involvement of lamins in the mechanistic basis of CIN induction. Lamin B2 localizes outside the spindle poles during mitosis and has a critical role in preventing CIN in colorectal cancers by maintaining spindle pole stability and spindle assembly (20). We surmise that Twist1-mediated decrease in lamin levels is an indirect means of contributing to CIN in colorectal cancers. It is well established that lamins maintain structural and functional integrity of the nucleus in eukaryotic cells (58,59). In addition, lamin loss affects chromatin organization and gene expression across cell types (19), while decreased levels of lamin A/C are also associated with cancers (60,61). Therefore, decrease in nuclear lamins through Twist1 overexpression may impact chromatin organization and gene expression. Stem cells and undifferentiated cells are characterized by relatively reduced lamin levels and ‘floppy’ chromatin (62). Furthermore, the increase in lamin levels correlates with differentiation (63,64). Excess levels of Twist1 and the concomitant reduction in lamin levels may induce stemness in transformed cells and create ‘founder’ populations of cancer stem cells, with elevated genomic instability and resilient sub-populations of cancer cells (65). This is consistent with a marked increase in Twist1 levels across cancers as well as in the aggressive colorectal cancers inferred from TCGA patient datasets (Fig. 6A and B).

The finding that Twist1 overexpression consistently downmodulates levels of checkpoint regulators further underscores the role of Twist1 in aggravating CIN in cancers (Fig. 4) (44). While reduced levels of lamins and checkpoint factors independently induce CIN, the underlying mechanisms of how lamins crosstalk with regulators of CIN remain unanswered. Notwithstanding a striking downregulation of the checkpoint factors at the transcript (Supplementary Material, Fig. S4B–F) and protein levels (Fig. 4), CIN− (DLD1) or the CIN+ (SW480) colorectal cancer cells nevertheless resist an increase in their overall ploidy levels upon Twist1 overexpression (Fig. 2E, G–H).

Twist1 overexpression also induces EMT in human mammary epithelial cells (HMLE) (66). Analyses of Twist1 occupancy from ChIP-Seq datasets available from HMLE cells (66) shows Twist1 enrichment on E-cadherin (CDH1 gene) at −29 kbp, +20 bp and +49 kbp from the TSS (Supplementary Material, Table S5). However, Twist1 does not show promoter occupancy on kinetochore associated genes that we examined. Gene ontology analysis of genes that show promoter binding (−1 to +1 kb from TSS) of Twist1 enriched for the p53 signaling pathway (Supplementary Material, Fig. S6A) (67). Interestingly, genes of the p53 pathway—MDM2, CHEK2 and CCNB1—show Twist1 occupancy on their respective promoters (Supplementary Material, Fig. S6B). Thus, Twist1 potentially modulates the p53 signaling pathway via MDM2, CHEK2 and CCNB1, suggestive of Twist1-dependent and Twist1-independent transcriptional modulation of genes that maintain chromosomal stability (Supplementary Material, Fig. S6, Table S5). Furthermore, Twist1 showed a relatively proximal occupancy to the TSS of the Lamin A/C and Lamin B2 promoters, consistent with their repression upon Twist1 overexpression (Supplementary Material, Table S5). Since Twist1 is also enriched at diverse distant sites with respect to the TSS, this suggests a hitherto undiscovered role of Twist1 in the regulation of long-distance chromatin interactions that further impinge on chromosomal and genomic stability in cancer cells.

Interestingly, analyses of transcription factor (TF)-gene interactions from ChIP-Seq data further implies that Twist1 potentially modulates the occupancy of histone modifiers (EZH2 and SUZ12) and transcription factors (SP3 and CTBP2) that collectively impinge on the factors regulating CIN and DNA damage, in the context of EMT (Supplementary Material, Fig. S5B).

Twist1 overexpression was associated with numerical chromosomal gains and losses and an increase in cellular heterogeneity with sub-populations consisting of both CIN− and CIN+ cells (Fig. 2F–H). Of note, the status of p53 is an important determinant of CIN regulation in colorectal cancers since DLD1 and SW480 cells with mutant p53 manifest CIN, while HCT116 cells, wild type for p53, do not show CIN upon Twist1 overexpression (Supplementary Material, Fig. S3). This contrasts with MCF7 breast cancer cells (wild type for p53) (25) which show CIN upon Twist1 overexpression (13). It is noteworthy that Twist1 overexpression showed a decrease in the levels of mutant p53 protein (Fig. 3K and L), the significance of which in the context of CIN is unclear. Interestingly, analyses of protein–protein interaction networks also reveal the impact of Twist1 and its interactors that impinge on the p53 signaling pathway (Supplementary Material, Figs S5A and S6). Taken together, these evidences imply p53-dependent and p53-independent regulation of CIN upon Twist1 overexpression in the context of the genetic background of cancers of diverse origin.

The observed increase in DNA double strand breaks marked by a higher frequency of γH2AX foci upon Twist1 overexpression is a case in point that further corroborates the increase in sub-chromosomal deletions, as revealed by array CGH analyses (Fig. 3A–J). Furthermore, DNA double strand breaks are precursors of chromosomal missegregation events, which potentially contribute to Twist1 induced CIN (68,69). Of note, both the colorectal cancer cell lines show amplifications and deletions with a higher preponderance of sub-chromosomal deletions (Fig. 3D). Genomic instability may further drive phenotypic switching between epithelial and mesenchymal fates by deletions and amplifications of genes associated with these two cell states (70). We speculate that Twist1-induced genome instability potentially drives EMT and therefore cancer progression. Furthermore, while Twist1 overexpression shows a positive correlation with the expression levels of EMT genes, on the contrary, it shows a cancer-specific correlation with DNA DSBs or CIN-associated genes (Fig. 6C).

**Figure 7:**
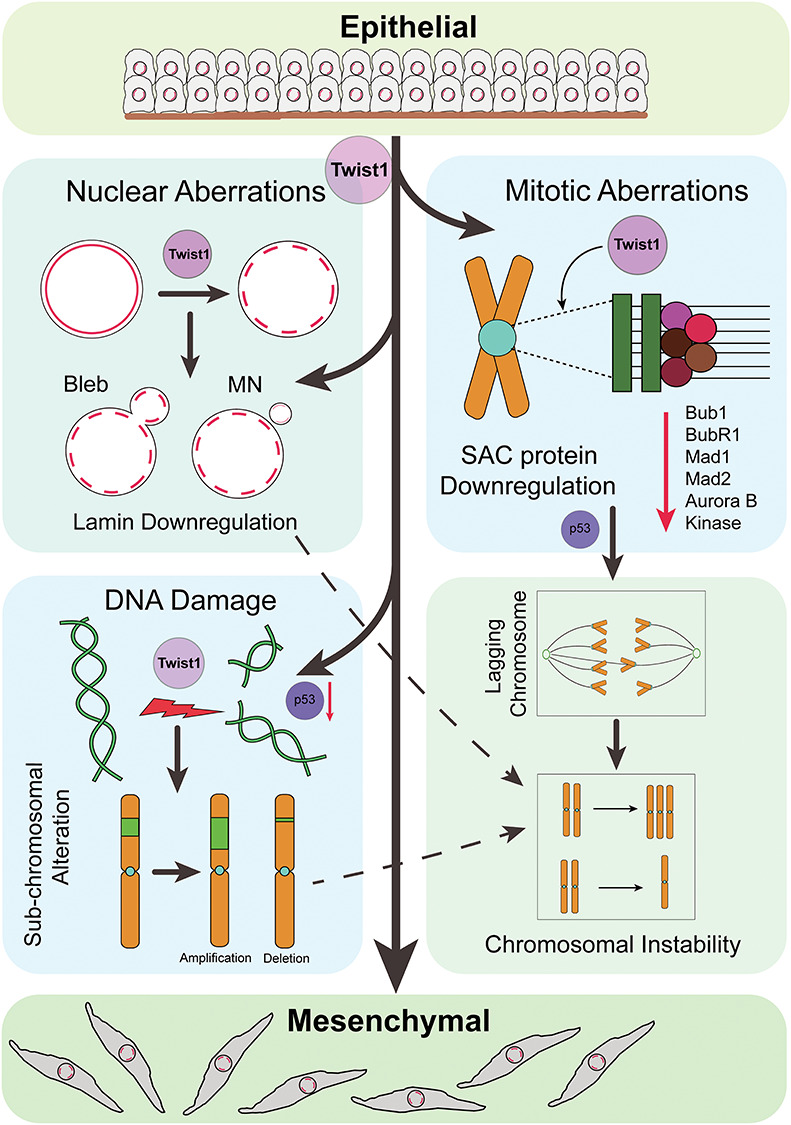
Speculative model suggesting a novel role for Twist1 overexpression in inducing CIN in colorectal cancer cells during EMTs. Twist1 overexpression in colorectal cancer cell lines (**i**) induces EMT, (**ii**) downregulates nuclear envelope proteins Lamin A/C, B1 and B2, associated with nuclear aberrations and CIN, (**iii**) induces DNA double strand breaks that result in enhanced sub-chromosomal alterations and CIN, potentially via p53 and (**iv**) downregulates cell cycle regulators Bub1, BubR1, Mad1, Mad2 and Aurora B Kinase leading to mitotic defects that contribute to enhanced CIN. In summary, Twist1 overexpression enhances CIN in the context of EMTs, which further contributes to cellular heterogeneity and cancer progression.

Taken together, these studies suggest that in addition to inducing EMT, Twist1 also enhances nuclear and mitotic aberrations and DNA double strand breaks that further contribute to genomic instability (Fig. 7). This is largely mediated by a collective decrease in levels of key checkpoint and genomic stability factors, underscoring the mechanistic involvement of Twist1 with CIN during EMTs.

## Materials and Methods

### Cell line validation

Human colorectal adenocarcinoma cell lines DLD1 and SW480 cells were validated by karyotyping and were kind gifts from the laboratory of Thomas Ried (NCI/NIH, Bethesda, USA). HCT116 colorectal cancer cells were from Mayurika Lahiri, IISER-Pune. The karyotypes of these cell lines are stable as they did not vary across passages. This was validated by analyses of metaphase spreads across passages that consistently showed a modal number of 45–46 chromosomes for the DLD1, 42–43 modal number chromosomes for HCT116 cells and a modal number of 56–57 chromosomes for the SW480 cells (Supplementary Material, Fig. S1A–D). These cells were free of mycoplasma contamination.

### Cell culture and transfection

DLD1 cells were cultured in RPMI1640 (Gibco, 11875), while HCT116 and SW480 were cultured in DMEM (Gibco, 11995) media, supplemented with heat inactivated 10% FBS (Gibco, 6140) and penicillin (100 units/ml)/Streptomycin (100 μg/ml) (Gibco, 15070-063). Cells were maintained in 5% CO_2_ at 37°C. DLD1 and SW480 and HCT116 cells (~0.4 × 10^6^) were transfected with ~2 μg of pBp-mTwist1 vector (Gift from Annapoorni Rangarajan, IISc Bengaluru, India and Robert Weinberg, MIT, USA) using LTX and PLUS (Invitrogen 15338100) reagents, with pBp-Empty vector as control. Cells were transfected with Twist1 for 24 h, and 1 μg/ml and 0.8 μg/ml puromycin (Gibco A11138), respectively, were added to select for transfected cells and cultured for another 48 h. All experiments were performed for 72 h.

### Western blotting

Protein lysates were prepared by scraping cells in ice cold RIPA buffer (pH = 7.2, 50 mm Tris Cl, 150 mm NaCl, 0.1% SDS, 0.01% sodium azide, 0.5% sodium deoxycholate, 1 mm DTT, 1% NP40) containing 1X protease inhibitor cocktail (Roche, 4693116001). This was followed by centrifugation at 300 *g* at 4°C for 10 min. Protein estimation was performed using BCA kit (Thermo, Pierce) and an equal amount of protein was loaded onto an SDS-PAGE gel. Proteins thus resolved were transferred onto a PVDF membrane (Millipore). Immunoblots were blocked using 5% non-fat milk prepared in 1× TBST (pH 7.4). Immunodetection was performed by adding primary antibodies against Twist1 (ab50887), 1:500; E-cadherin (ab1416), 1:1000; Vimentin (Sigma, V2258), 1:500; Lamin (A + C) (ab108595), 1:1000; Lamin B1 (ab16048), 1:1000; Lamin B2 (ab8983), 1:500; Bub1 (ab54893), 1:1000; BubR1 (ab54894), 1:1000; Mad1 (ab126148), 1:3000; Mad2 (ab24588), 1:500; Aurora B Kinase (CST3094), 1:1000; Aurora B Kinase (ab2254), 1:1000; p53 (ab28) 1:1000; GAPDH (Sigma, G9545), 1:10 000. Secondary antibodies used were sheep anti-mouse-HRP (Amersham, NA9310V), 1:10 000; donkey anti-rabbit-HRP (Amersham, NA9340V) and goat anti-rat-HRP (Amersham, NA935) 1:10 000, for 1 h at RT. Between incubation, blots were rinsed thrice with 1× TBST for 10 min each at RT. Chemiluminescent substrate ECL Prime (Amersham, 89168-782) was used to develop immunoblots and imaged with ImageQuant LAS4000.

### Immunofluorescence assay

~ 0.4 × 10^6^ cells/well were seeded onto glass coverslips and transfections were performed as described previously. Cells were washed with 1× PBS and fixed with 4% paraformaldehyde (Sigma, P6148) prepared in 1× PBS, pH 7.4 at RT for 10 min, washed thrice in 1× PBS (5 min each). Fixation was followed by permeabilization in 0.5% Triton-X-100 prepared in 1× PBS at RT for 10 min. Cells were blocked in 1% bovine serum albumin (BSA) (Sigma, A2153) prepared in 1× PBS, for 30 min and washed three times with 1× PBS. Incubation with primary antibodies was performed in 0.1% BSA for 90 min at RT and with secondary antibodies for 60 min at RT, with washes in between using 1× PBS. Primary antibodies used were E-cadherin (ab1416) 1:500; Phalloidin Alexa Fluor 488 (A12379) 1:100; Lamin A (ab26300) 1:1000; γH2AX (ab26350) 1:750. Secondary antibodies were diluted in 1× PBS + 0.1% Triton X-100 (PBST): Goat anti Rabbit-Alexa 488 (Invitrogen, A11034), 1:1000; Goat anti Rabbit-Alexa 568 (Invitrogen, A11011), 1:1000; Goat anti mouse-Alexa 488 (Invitrogen, A11029), 1:1000; Goat anti mouse-Alexa 568 (Invitrogen, A11004), 1:1000. Cells were washed thrice in 1× PBST. Cells were counterstained with 4′,6-diamidino-2-phenylindole (DAPI) (Invitrogen, D1306) for 2 min at RT, washed in 1× PBS for 5 min and mounted in Slowfade Gold Antifade (Invitrogen, S36937). Cells were imaged on a Zeiss LSM710 confocal microscope with 405, 458 and 561 nm laser lines, using a 63× oil immersion objective, NA 1.4 at 1× digital zoom. X–Y resolution was 512 × 512. Confocal z-stacks were collected at intervals of 0.34 μm.

### Flow cytometry

The empty vector and Twist1 transfected DLD1 and SW480 cells were trypsinized, washed with 1× PBS and then fixed in chilled 70% ethanol. Ethanol was added dropwise to the pellet while vortexing. This ensured fixation of all cells and minimized clumping. After chilling on ice for 15 min, the cells were centrifuged at 200 *g* for 10 min. The pellet was resuspended in 1× PBS, subjected to RNase (Sigma) (10 μg) treatment at 37°C for 45 min. Further, propidium iodide (Sigma) (10 μg) was added to the samples. Cell suspensions were subsequently run on FACSCalibur (BD Biosciences) and analyzed using Cell Quest Pro software.

### Metaphase spread preparation

Colcemid (Roche) (1% v/v) was added to cells (empty vector, Twist1 transfected) at ~60–70% confluency and incubated for 90 min at 37°C. The media was collected, and the cells were washed with 1× DPBS and trypsinized. Cells were centrifuged at 200 *g* at 4°C for 10 min. The pellet was resuspended in 4 ml pre-warmed 0.075 M KCl and incubated at 37°C for 30 min. ~4–5 drops of fixative [methanol:acetic acid (3:1)] were added, and cells were centrifuged at 200 *g* at 4°C for 10 min. The supernatant was discarded, followed by two more washes in fixative. Cells were finally resuspended in ~100–200 μl of fixative as per the volume of the pellet, followed by dropping on clean glass slides.

### Array comparative genomic hybridisation (array CGH)

High-quality genomic DNA was extracted from DLD1 and SW480 cells transfected with pBp-Empty as a control and pBp-mTwist1. DNA was fragmented using restriction digestion. The control sample was labelled with Cy3 and Twist1 samples using Cy5. The DNA was hybridized on the Agilent Human 1X1M array (Agilent 073558). Image analysis was performed using Agilent Feature Extraction and Agilent CytoGenomics 3.01.1 software. Copy number alterations (CNAs) were mapped to the genome build GRCh38/hg19 for analysis and interpretation. Detailed protocol for array CGH is provided as Supplementary Information S1. We acknowledge Genotypic Technology Private Limited, Bengaluru, India for sample processing and data analysis.

### RT-PCR

Total RNA was extracted using the Trizol method (71) from DLD1 and SW480 cells transfected independently with vector control and Twist1. cDNA was synthesized from 1 μg of total RNA with the Verso cDNA kit (AB-1453/B) using Olido(dT) primers. cDNA was used as a template, and RT-PCR was carried out using primers designed to span intron-exon junctions (Supplementary Information S2). GAPDH was used as internal control. Real-time quantitative PCR was performed in 5 μl reaction mixture containing KAPA SYBR FAST qPCR Master Mix (2×) (KK4602, Merck) and 2 μm each of the forward and reverse primer using the Bio-Rad RT-PCR instrument (CFX96 Touch). Fold change in expression was calculated by double normalization of Ct values to the internal control (GAPDH) and empty vector control by the 2^−ΔΔ*Ct*^ method (72).

### Statistical analysis and graphs

A minimum of 30 cells were analyzed for each biological replicate. All experiments were performed in at least *N* = 2 independent biological replicates. The number of technical replicates (*n*) differs for each experiment. Statistical analysis was performed, and graphs were plotted with GraphPad Prism 6 software.

### Image processing and analysis

Images were quantified using ImageJ software. E-cadherin levels were measured by tracing out E-cadherin staining manually, and intensity was measured along the traced line. For actin staining, aspect ratio was calculated as a ratio of major axis/minor axis. For analyses of γH2AX foci, thresholding was performed for each nucleus counterstained with DAPI, and the ‘find maxima’ function was used to enumerate the number of γH2AX foci per nucleus.

### Mathematical modeling

The network was simulated using the tool ‘RAndomized CIrcuit PErturbation (RACIPE)’ (Supplementary Information S3) (54). RACIPE models a given regulatory network using a system of Ordinary Differential Equations (ODEs) and samples multiple parameter sets randomly via a uniform distribution from a predefined range of parameters. For each parameter set, the system of ODEs representing the interactions in the network is simulated at multiple initial conditions to identify the number of steady states. For the current analysis, 10 000 parameter sets were sampled, and 100 random initial conditions were chosen for each parameter set. The ODEs were integrated using Euler’s method of numerical integration. Linear regression was used to fit the gene expression data obtained from RACIPE to a line. Corresponding *P-*value ranges are reported.

### TCGA expression analysis

Gene expression (RSEM gene-normalized, version 2016_01_28) and somatic mutation data (MC3) of TCGA samples (*n* = 8657) across 30 tumor types were downloaded from Firebrowse server (http://firebrowse.org). The correlation coefficient between TWIST1 expression and other gene expressions/mutation burden/copy number alterations and its significance were computed using iteratively reweighted least-squares approach. To adjust for multiple hypothesis testing, Bonferroni correction on *P* values per gene set was performed, and *q* < 0.01 was considered as significant. All plots were generated using the Seaborn package in Python. The results shown here are in whole or part based upon data generated by the TCGA Research Network: https://www.cancer.gov/tcga.

## Supplementary Material

Supplementary_Figures_ddaa076

Supplementary_Information_ddaa076

Supplementary_Uncropped_blots_ddaa076

Suppl_Files_ddaa076
