# Supplementary material for: Twist1 induces chromosomal instability (CIN) in colorectal cancer cells
Source: Hum Mol Genet. 2020 Apr 27;29(10):1673–88. doi: 10.1093/hmg/ddaa076 (PMC7322571; doi:10.1093/hmg/ddaa076)
Supplement: Supplementary_Figures_ddaa076 [file supplementary_figures_ddaa076.pdf]

Supplementary Figures

Figure S1

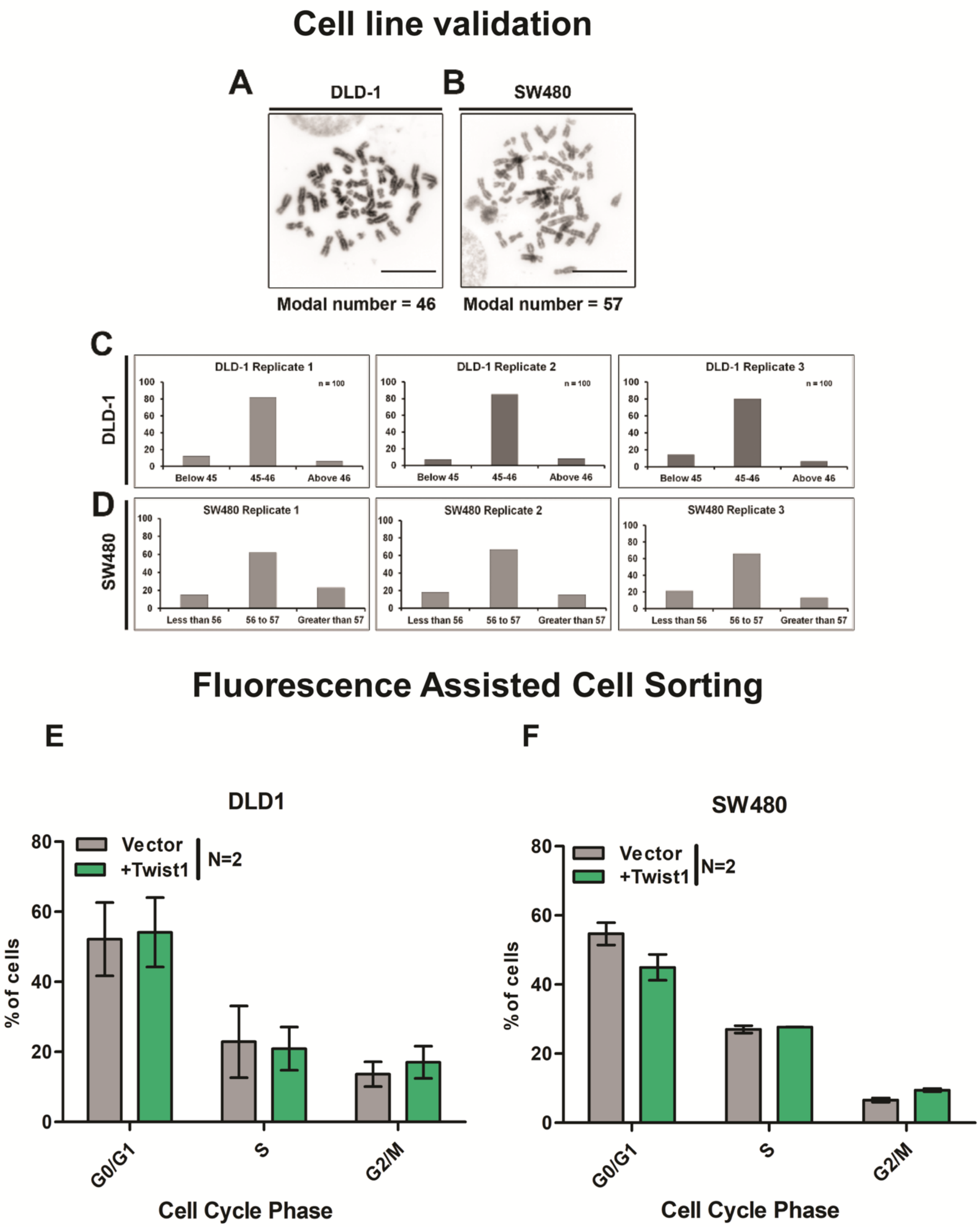

Figure S1: Cell line validation by chromosome counting and FACS

(A-B) Representative metaphase spreads of colorectal cancer cell lines DLD1 and SW480. **(C)** Quantification of chromosome numbers from metaphase spreads showing modal chromosome numbers of 44-45 for DLD1 cells. Data from N=3 independent biological replicates. Scale bar ~10 μm. **(D)** Quantification of chromosome numbers from metaphase spreads showing modal chromosome number of 56-57 for SW480 cells. Data from N=3 independent biological replicates. **(E-F)** Quantification of distribution of cells across cell cycle phases from Fluorescence Assisted Cell Sorting shows no significant effect of Twist1 overexpression on cell cycle. Data quantified from N=2 independent biological replicates (N=2, Mean with Range).

Figure S2 Nuclear and Mitotic Aberrations

A

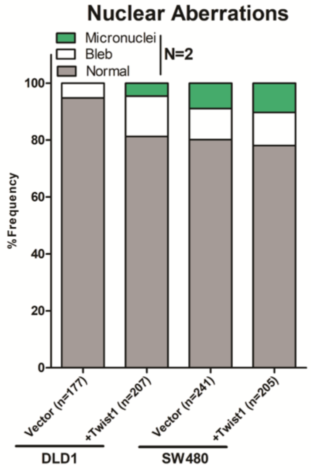

B

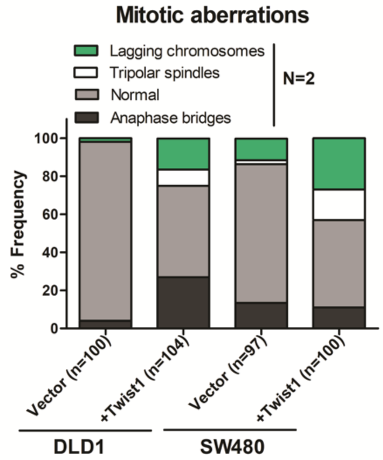

Table S1

	DLD1		SW480	
	Vector	+Twist1	Vector	+Twist1
Normal	95%	81%	80%	78%
Blebs	5%	14%	11%	12%
Micronuclei	0%	5%	9%	10%

Table S2

	DLD1		SW480	
	Vector	+Twist1	Vector	+Twist1
Lagging Chromosomes	2%	16%	11%	27%
Anaphase Bridges	4%	27%	14%	11%
Tri-polar Spindles	0%	9%	2%	16%
Normal Anaphases	94%	48%	73%	46%

C

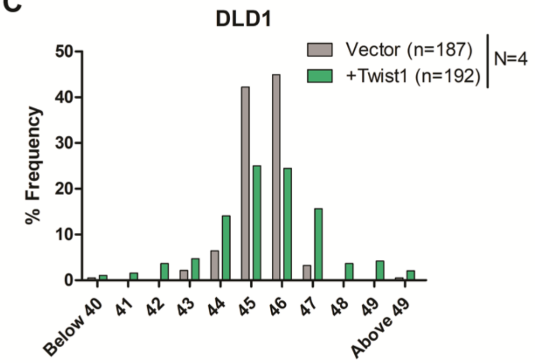

D

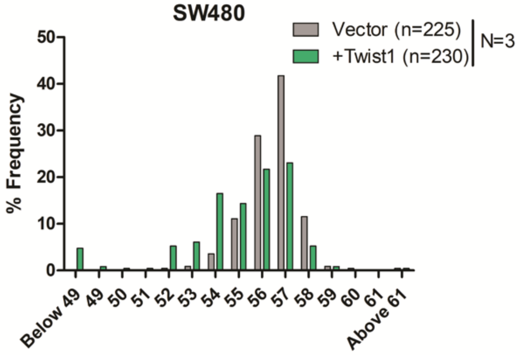

Table S3

	DLD1			SW480		
	<45	45-46	>46	<56	56-57	>57
Vector	9%	87%	4%	16%	71%	13%
+Twist1	25%	49%	26%	49%	45%	6%

E

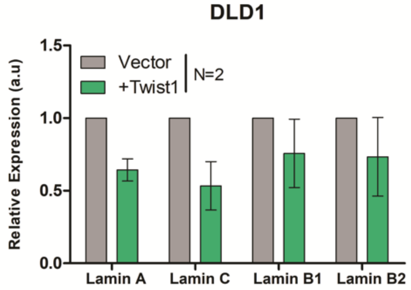

F

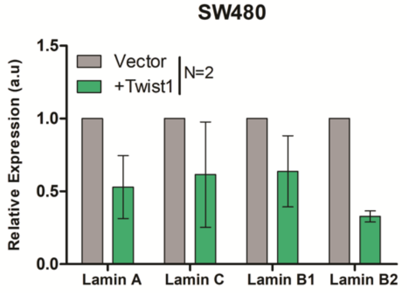

Figure S2: Nuclear and Mitotic Aberrations

(A) Quantification of nuclear aberrations plotted with frequency of normal cells observed across both cell lines (N=2, Mean). **(B)** Quantification of mitotic aberrations plotted with frequency of normal cells observed across both cell lines (N=2, Mean). **(Table S1)** Table representing the average % frequency changes in nuclear aberrations observed upon Twist1 overexpression across cell lines. Upon Twist1 overexpression, the % frequency of normal cells decreases, while the % frequency of blebs and micronuclei increases across cell lines. Data quantified from two independent biological replicates (N=2, Mean). **(Table S2)** Table representing the average % frequency changes in mitotic aberrations observed upon Twist1 overexpression across cell lines. Upon Twist1 overexpression, the % frequency of normal cells decreases, while the % frequency of anaphase bridges, lagging chromosomes and tripolar spindles increases in DLD1 cells. The % frequency of anaphase bridges decreases, while that of lagging chromosomes and tripolar spindles increases in SW480 cells. Data quantified from two independent biological replicates (N=2, Mean). **(C)** Representative histograms of chromosome count upon Twist1 overexpression across DLD1 cell line. Data quantified from n>180 independent metaphase spreads collected from four independent biological replicates (N=4). **(D)** Representative histograms of chromosome count upon Twist1 overexpression across SW480 cell line. Data quantified from n> 200 independent metaphase spreads collected from three independent biological replicates (N=3). **(Table S3)** Table representing the % frequency of gains and losses as compared to normal cells upon Twist1 overexpression. **(E-F)** Quantification of lamin levels upon Twist1 overexpression in **(E)** DLD1 **(F)** SW480**, (Fig.2I).** Quantification of band intensities from two independent biological replicates, normalized to GAPDH (N=2, Mean with Range).

Figure S3

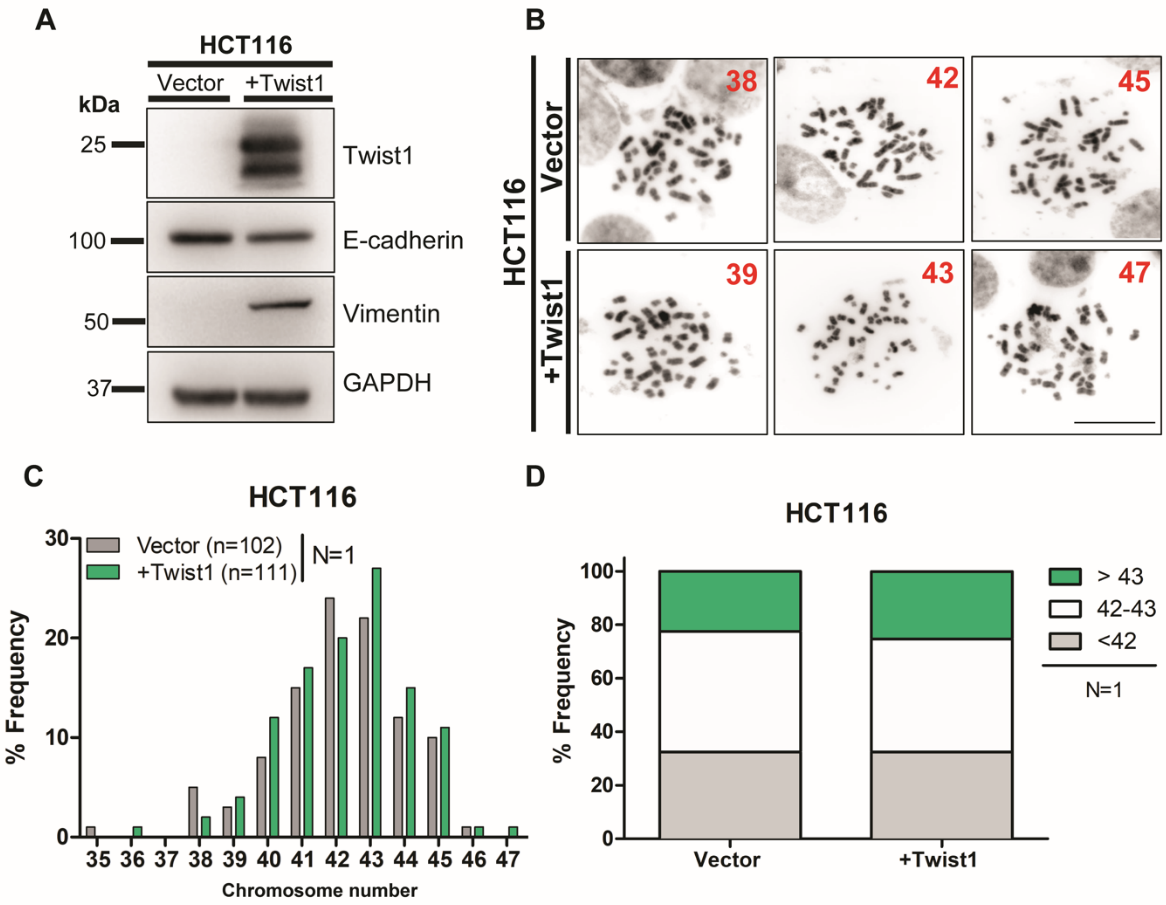

Table S4

	<42	42-43	44-45	<45
**Vector**	32%	45%	22%	1%
**+Twist1**	32%	42%	23%	2%

Figure S3: Twist1 overexpression in HCT116 colorectal cancer cells

(A) Representative immunoblot showing EMT induction upon Twist1 overexpression. E-cadherin shows a marginal decrease, while Vimentin shows a concomitant increase in levels in HCT116 cells. **(B)** Representative images of metaphase spreads derived from HCT116 upon Twist1 overexpression. Scale bar ~20 μm. **(C)** Histogram showing quantification of metaphase spreads derived from cells treated with vector control (N=1, n=102) and Twist1 overexpression respectively (N=1, n=111). **(D)** Quantification of whole chromosomal gains and losses in HCT116 cells. (Data quantified from n=100 independent metaphase spreads collected from N=1 single experiment). **(Table S4)** Table representing percentage frequency of whole chromosomal gains and losses in HCT116 cells upon Twist1 overexpression.

Figure S4

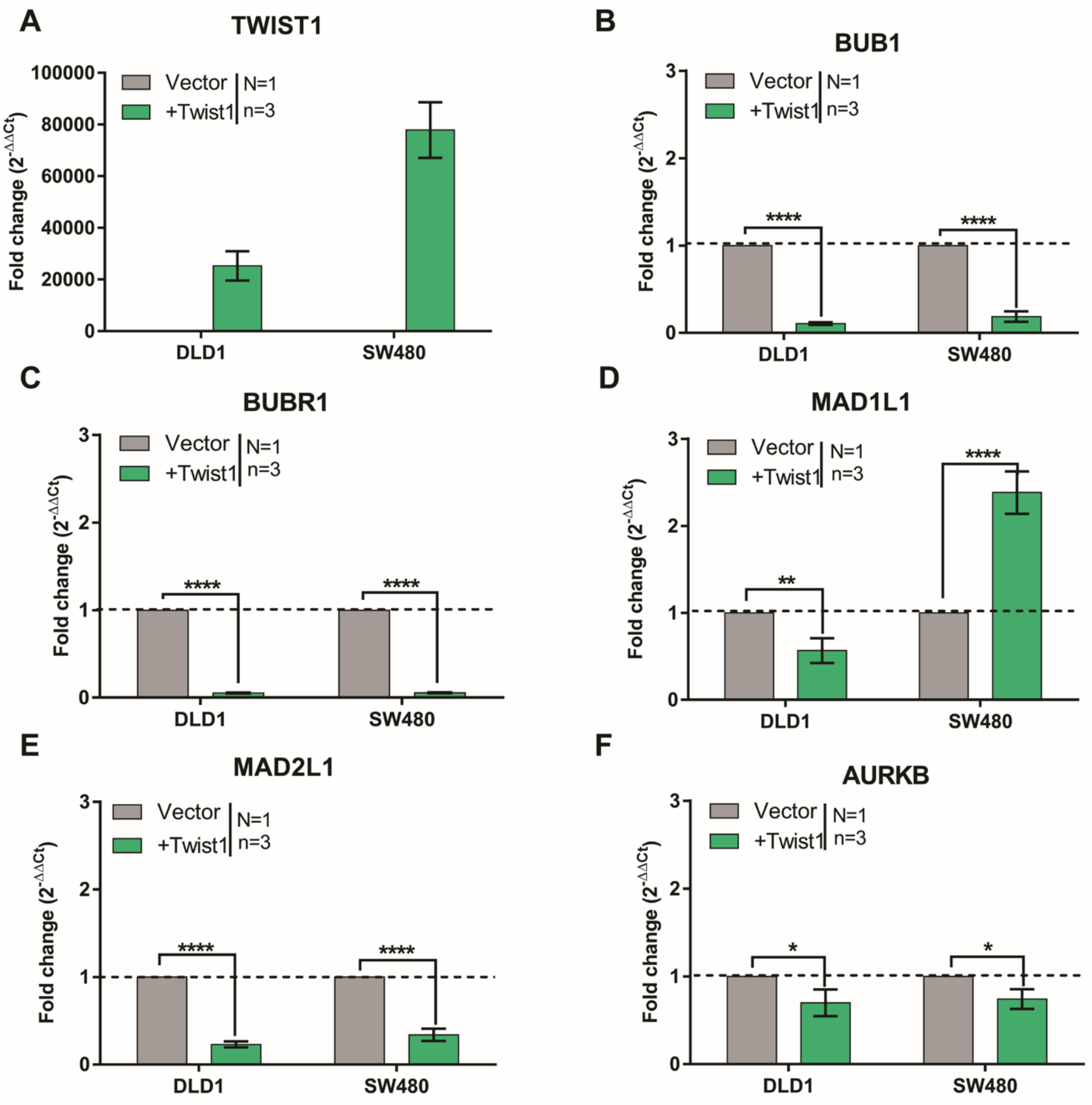

A
TWIST1
Vector
+Twist1
N=1
n=3
Fold change ($2^{-\Delta\Delta Ct}$)
100000
80000
60000
40000
20000
0
DLD1
SW480
B
BUB1
Vector
+Twist1
N=1
n=3
Fold change ($2^{-\Delta\Delta Ct}$)
****
****
DLD1
SW480
C
BUBR1
Vector
+Twist1
N=1
n=3
Fold change ($2^{-\Delta\Delta Ct}$)
****
****
DLD1
SW480
D
MAD1L1
Vector
+Twist1
N=1
n=3
Fold change ($2^{-\Delta\Delta Ct}$)
**
****
DLD1
SW480
E
MAD2L1
Vector
+Twist1
N=1
n=3
Fold change ($2^{-\Delta\Delta Ct}$)
****
****
DLD1
SW480
F
AURKB
Vector
+Twist1
N=1
n=3
Fold change ($2^{-\Delta\Delta Ct}$)
*
*
DLD1
SW480

Figure S4: Effect of Twist1 overexpression on transcript levels of checkpoint genes

(A) Gene expression levels determined by RT-PCR of Twist1 upon Twist1 overexpression in DLD1 and SW480 cells, normalized to vector control. **(B-F)** Gene expression profiling of checkpoint genes determined by RT-PCR for checkpoint genes upon Twist1 overexpression in DLD1 & SW480. Data normalised to its respective vector control. (Data quantified from N=1 single experiment that include n=3 independent technical replicates, unpaired t-test, Mean±SD, *P < 0.05, **P < 0.01, ***P < 0.001 and ****P < 0.0001).

Figure S5

A

Protein- Protein interactions

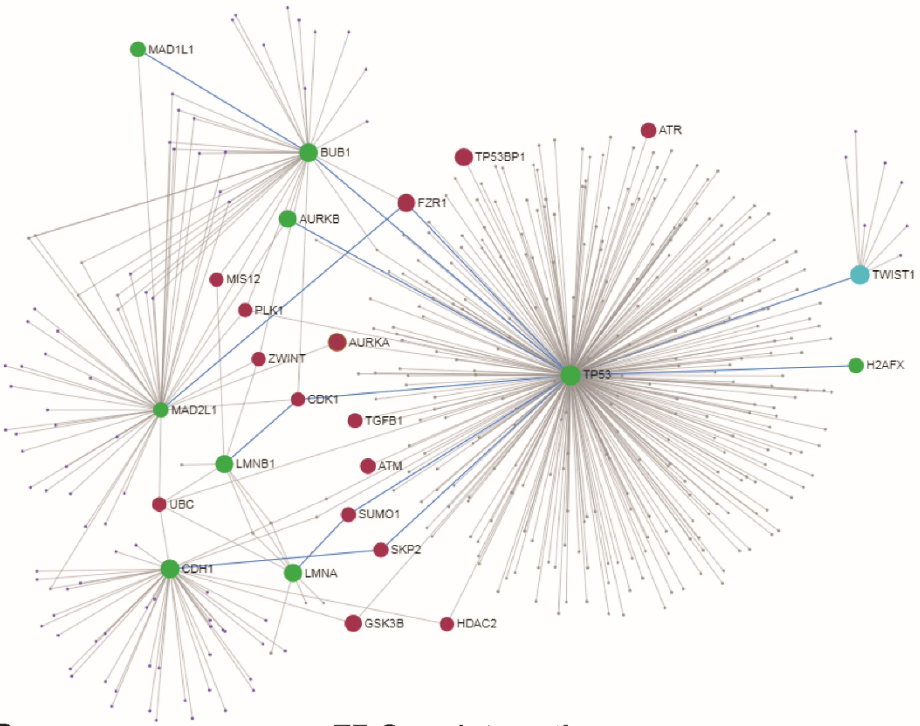

B

TF-Gene interactions

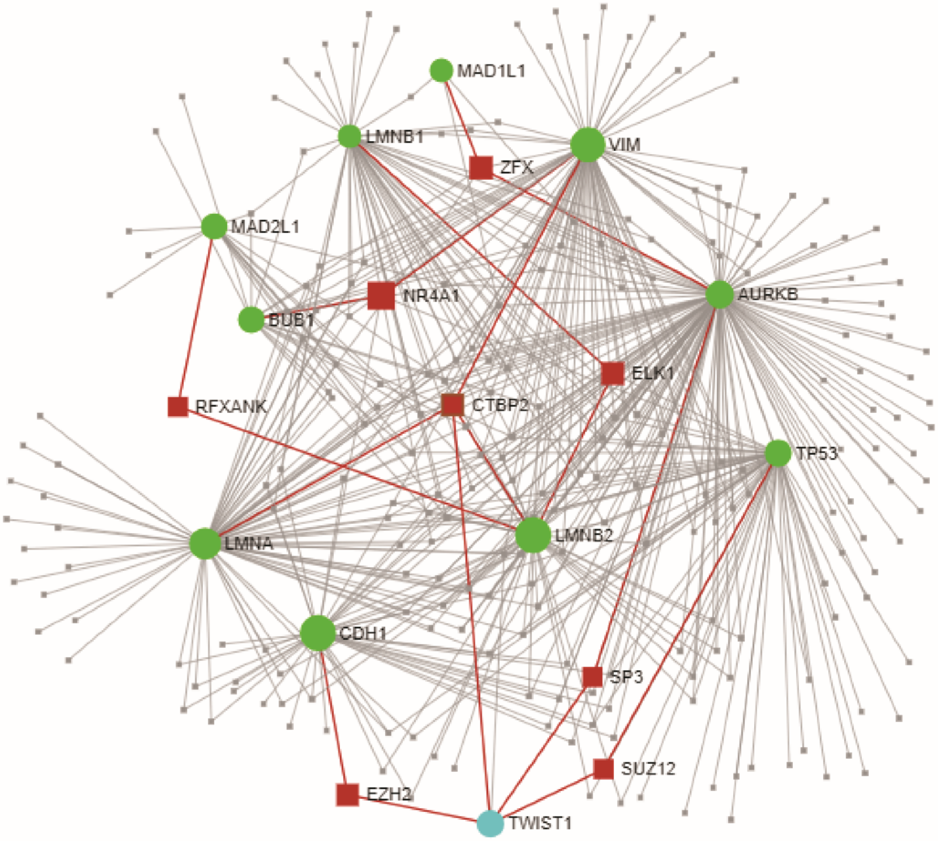

Figure S5: Correlation between levels of Twist1, EMT and CIN factors

(A) Generic Protein-Protein interactions of Twist1 & other proteins examined in this study (E-cadherin, Vimentin, Bub1, BubR1, Mad1, Mad2, Aurora B kinase, p53, LaminA/C, LaminB1, LaminB2) generated using NetworkAnalyst. Blue line: Candidate and key protein-protein interactions in the context of Twist1, EMT and CIN factors. Gray line: All other protein-protein interactions. ● Candidate EMT and CIN factors reported in this study

● Potential protein-protein interactors connecting ● Twist1 with EMT, CIN and DNA damage.

(B) Transcription factor-Gene interactions between TWIST1 & other target genes (CDH1, VIM, BUB1, MAD1L1, MAD2L1, AURKB, TP53, LMNA, LMNB1 and LMNB2) examined in this study generated using NetworkAnalyst. ● TWIST1 ● Input candidate genes ■ transcription factor enrichment on genes derived from ENCODE ChIP-seq data.

Table S5 **Analysis of Twist1 occupancy from ChIP seq data of HMLE cells**

Gene Category	Gene Name	Occupancy	Chromosome	Start	End	Annotation	Distance to TSS (bp)
EMT	CDH1	+	Chr.16	67298818	67299408	Intergenic	-29583
	CDH1	+	Chr.16	67328548	67328884	promoter-TSS (NM_004360)	+20
	CDH1	+	Chr.16	67377982	67378379	intron (NM_004360, intron 2 of 15)	+49484
	VIM	-	Chr.10	N.A.	N.A.	N.A.	N.A.
Checkpoint	BUB1	-	Chr.2	N.A.	N.A.	N.A.	N.A.
	BUBR1	-	Chr.15	N.A.	N.A.	N.A.	N.A.
	MAD1L1	+	Chr.7	2201955	2202527	intron (NM_001013836, intron 10 of 18)	+36868
	MAD1L1	+	Chr.7	2122201	2122623	intron (NM_001013836, intron 11 of 18)	+116697
	MAD2L1	+	Chr.4	121632168	121632386	Intergenic	-424816
	AURKB	-	Chr.17	N.A.	N.A.	N.A.	N.A.
	TP53	-	Chr.17	N.A.	N.A.	N.A.	N.A.
Nuclear Envelope	LMNA	+	Chr.1	154339840	154340272	intron (NM_001282625, intron 3 of 12)	-11029
	LMNA	+	Chr.1	154360004	154360693	intron (NM_005572, intron 1 of 9)	-2184
	LMNA	+	Chr.1	154365724	154366780	intron (NM_001282624, intron 2 of 10)	+3720
	LMNA	+	Chr.1	154355937	154356303	intron (NM_005572, intron 1 of 9)	+5035
	LMNB1	-	Chr.5	N.A.	N.A.	N.A.	N.A.
	LMNB2	+	Chr.19	2404030	2404390	intron (NM_032737, intron 1 of 11)	+3756

Table S5 ChIP-Sequencing analyses of Twist1 occupancy from HMLE (human mammary epithelial) cells upon Twist1 overexpression.

Figure S6

A

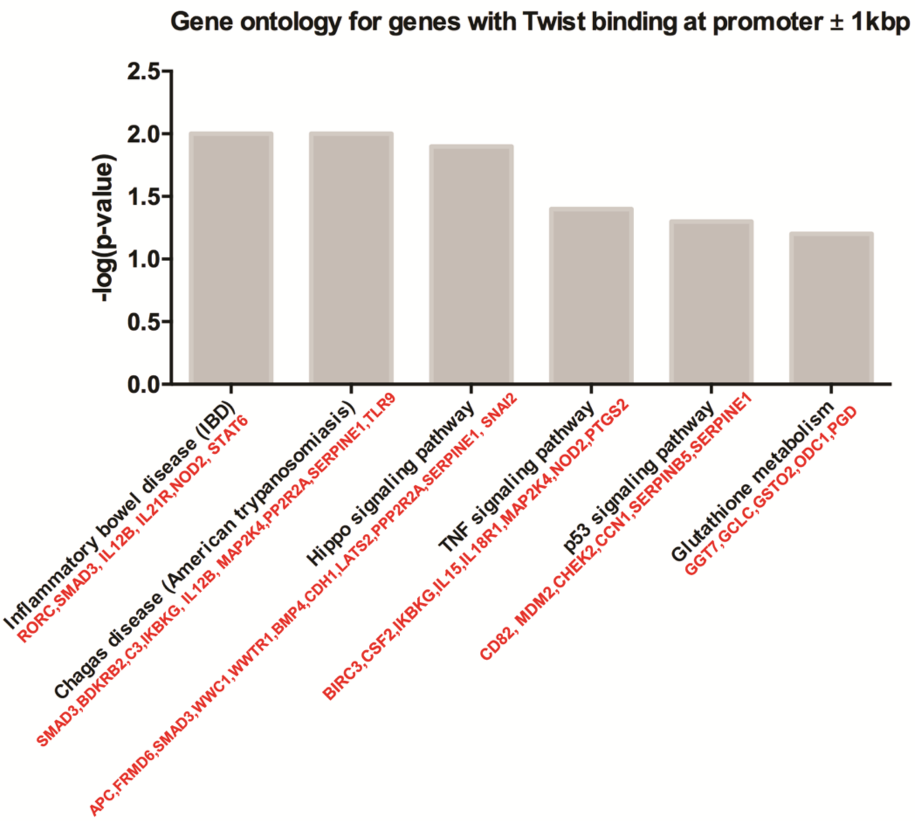

Gene ontology for genes with Twist binding at promoter ± 1kbp
-log(p-value)
2.5
2.0
1.5
1.0
0.5
0.0
Inflammatory bowel disease (IBD)
RORC,SMAD3, IL12B, IL21R,NOD2, STAT6
Chagas disease (American trypanosomiasis)
SMAD3,BDKRB2,C3,IKBKG, IL12B, MAP2K4,PP2R2A,SERPINE1,TLR9
Hippo signaling pathway
APC,FRMD6,SMAD3,WWC1,WWTR1,BMP4,CDH1,LATS2,PPP2R2A,SERPINE1, SNAI2
TNF signaling pathway
BIRC3,CSF2,IKBKG,IL15,IL18R1,MAP2K4,NOD2,PTGS2
p53 signaling pathway
CD82, MDM2,CHEK2,CCN1,SERPINB5,SERPINE1
Glutathione metabolism
GGT7,GCLC,GSTO2,ODC1,PGD

B

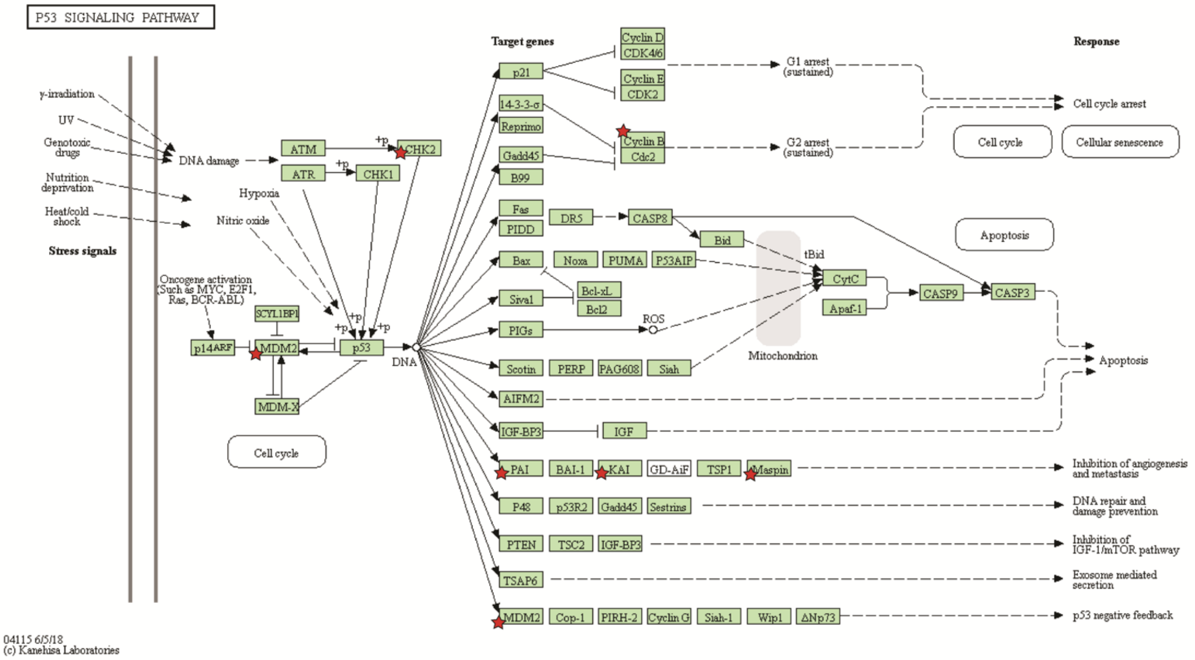

P53 SIGNALING PATHWAY
γ-irradiation
UV
Genotoxic drugs
Nutrition deprivation
Heat/cold shock
Stress signals
DNA damage
ATM
ATR
CHK2
CHK1
Hypoxia
Nitric oxide
Oncogene activation (Such as MYC, E2F1, Ras, BCR-ABL)
SCYL1BP1
p14ARF
MDM2
p53
MDM-X
DNA
Cell cycle
Target genes
p21
14-3-3-σ
Reprimo
Gadd45
B99
Cyclin D
CDK4/6
Cyclin E
CDK2
Cyclin B
Cdc2
G1 arrest (sustained)
G2 arrest (sustained)
Response
Cell cycle arrest
Cell cycle
Cellular senescence
Fas
PIDD
DR5
CASP8
Bid
tBid
Bax
Noxa
PUMA
P53AIP
Bcl-xL
Bcl2
Siva1
PIGs
ROS
CytC
Apaf-1
CASP9
CASP3
Mitochondrion
Apoptosis
Scotin
PERP
PAG608
Siah
AIFM2
IGF-BP3
IGF
PAI
BAI-1
KAI
GD-AiF
TSP1
Maspin
Inhibition of angiogenesis and metastasis
P48
p53R2
Gadd45
Sestrins
DNA repair and damage prevention
PTEN
TSC2
IGF-BP3
Inhibition of IGF-1/mTOR pathway
TSAP6
Exosome mediated secretion
MDM2
Cop-1
PIRH-2
Cyclin G
Siah-1
Wip1
ΔNp73
p53 negative feedback
04115 6/5/18
(c) Kanehisa Laboratories

Figure S6: Twist1 is enriched on promoters of genes in the p53 signaling pathway

(A) Gene Ontology analysis of genes that show Twist1 occupancy enriched within (-1kbp to +1kbp) of the promoter region using DAVID Bioinformatics Resources 6.8. **(B)** p53 pathway from KEGG. Red stars indicate genes of the p53 pathway showing Twist1 occupancy in their promoter region.
