# Supplementary material for: Twist1 induces chromosomal instability (CIN) in colorectal cancer cells
Source: Hum Mol Genet. 2020 Apr 27;29(10):1673–88. doi: 10.1093/hmg/ddaa076 (PMC7322571; doi:10.1093/hmg/ddaa076)
Supplement: Supplementary_Information_ddaa076 [file supplementary_information_ddaa076.pdf]

Supplementary Information (SI)

SI#1 Array Comparative Genomic Hybridisation (Fig. 3)

DNA Quality:

The DNA quality, purity and quantity control test was determined using Nanodrop-1000 (JH Bio, USA) followed by a gel QC.

Sample Labelling

To ensure high quality of aCGH data, Agilent Direct method has been used for the sample processing. About 1 ug of control and test DNA was used for the restriction digestion in the master mix containing *AluI* and *RsaI* restriction enzymes as per manufacturer's recommendation. The samples were incubated at 37°C for 2 hours followed by heat inactivation of enzymes at 65°C for 20 minutes. To confirm the efficiency of restriction enzymes to obtain fragments of size 200-500bp, about 2 μL of the digested gDNA was tested on a 0.8% agarose gel. Labelling of samples was done by random priming method, in which the random hexamers, Cy3-dUTP & Cy5-dUTP, dNTP, Buffer and Klenow enzyme was used. Briefly, 1X Random primer mix was added to each of 26μl digested control and test samples. The DNA was denatured at 95 °C for 3 minutes followed by snap chill on ice for 5 minutes. Master mix for Cy3 and Cy5 dNTPs was done separately to ensure that the control sample is labelled with Cy3 and test sample with Cy5 respectively. About 19 ul of labelling master mix prepared as per manufacturer's recommendation was added to the denatured control and test DNA sample and incubated at 37°C for 2 hours followed by enzyme heat inactivation at 65°C for 10 minutes. The labelled samples were cleaned up by Amicon 30kDa filter size exclusion filter. The sample volume was adjusted with respect to array format. The specific activity and yield was optimum to proceed for the hybridization.

Hybridization and Wash

Equal amount of labelled Test & Control DNA sample was added into a fresh tube containing 50 µl of Human Cot-1 DNA (1mg/ml), 52ul of Agilent 10X blocking agent and 260ul Agilent 2X hybridization buffer. The total hybridization volume was 520ul. The above hybridization mix was denatured at 95°C for 3 minutes and incubated the microfuge tubes at 37°C for 30 minutes.

The samples were hybridized at 65°C for 40 hours in the hybridization chamber. After hybridization, the slides were washed using aCGH Wash Buffer1 (Agilent Technologies, Part Number 5188-5221) at room temperature for 5 minutes and aCGH Wash Buffer 2 (Agilent Technologies, Part Number 5188-5222) at 37°C for 1 minute. The slides were then washed with Acetonitrile for 10 seconds. The microarray slide was scanned using Agilent Scanner (Agilent Technologies, Part Number G2565CA).

Microarray Data Analysis

Image analysis was performed using Agilent Feature Extraction software. Feature extracted raw data was normalized by applying LOWESS normalization method & further data analysis was carried out using Agilent CytoGenomics 3.01.1 software and excel. Agilent CytoGenomics supports CGH arrays, which allows the detection of regions of loss or gain. Aberration Detection Method II(ADM-2) algorithm was applied to identify significant regions having amplifications and deletions among each of the samples. GC Correction algorithm was then applied to correct aCGH log ratio data for the presence of "wavy" artefacts. Penetrance analysis was performed to find the percentage of samples that share aberrations in a particular genomic region among multiple samples (Amplification and deletions are considered separately). Common aberrations among the samples were identified. Differential aberration analysis for two groups was performed and specific aberration for each group was determined. Graphical representation has been done using

Human UCSC genome browser by loading the data in wiggle file format. Various Genome view chromosome view, Gene view plots were generated for the amplification and deletions data with respect to all samples.

SI#2 List of RT- PCR Primers for Twist1 and CIN genes (Fig S4)

No.	Gene Name	Sequence
1	TWIST1	F- GCGCTGGGGAAGATCATC
		R- GGTCTGAATCTTGCTCAGCTT
2	BUB1	F- TGTTGAGCAGGTTGTTATGTATTG
		R- GTCTGTCTTCATTTACCCATTGC
3	BUBR1	F- CAGCCAGTTATGACACCATGTA
		R- TGATGGCTCTGAACCCTTTG
4	MAD1L1	F- CCTTCAGACTTGGACTGTGTC
		R- CATGGTTGCTTTCGCGATTAC
5	MAD2L1	F- ACAGCTACGGTGACATTTCT
		R- GTCCCGACTCTTCCCATTT
6	AURKB	F- CATCGTCAAGGTGGACCTAAAG
		R- GGGTTATGCCTGAGCAGTTT
7	GAPDH	F- CGAGATCCCTCCAAAATCAAG

		R- GCAGAGATGATGACCCTTTTG

SI3. Mathematical analysis of network depicting the interactions among TWIST, EMT and CIN (Fig.5)

The network was simulated using the tool “RAndomized CIrcuit PErturbation (RACIPE)” (Huang et al. 2017), available for download at https://github.com/simonhb1990/RACIPE-1.0. Briefly, RACIPE models a given regulatory network using a system of Ordinary Differential Equations. Each equation in the system represents the dynamics of one node in the network and is of the following form:

$$\frac{dX_i}{dt} = g_{X_i} * \prod_{j=1}^{n} H^S(X_j, X_i) - k_{X_i} * X_i$$

Where g and k represent the production and degradation of a node and $H^S(X_j, X_i)$ is a modified hill function that represents the regulation of X_i by X_j. Further details are available in (Huang et al. 2017). The tool then samples multiple parameter sets randomly via a uniform distribution from a pre-defined range of parameters. As the exact kinetic parameters are often not available for biological systems, this sampling allows us to obtain a generic behaviour of the network, accounting for cell-cell variability in kinetic parameters. For each such parameter set, the system is simulated at multiple initial conditions to identify the number of steady states. For the current analysis, 10000 parameter sets were sampled, and 100 random initial conditions were chosen for each parameter set. The ODE’s were integrated using Euler’s method of numerical integration. All parameter ranges used in this study are defaults of the tool. Linear regression was used to fit

coupled gene expression data obtained from RACIPE to a line. Corresponding p-value ranges are reported.
