# Supplementary figures and images for: Twist1 induces chromosomal instability (CIN) in colorectal cancer cells
Source: Hum Mol Genet. 2020 Apr 27;29(10):1673–88. doi: 10.1093/hmg/ddaa076 (PMC7322571; doi:10.1093/hmg/ddaa076)

## UNCROPPED BLOTS

**Fig.1A**

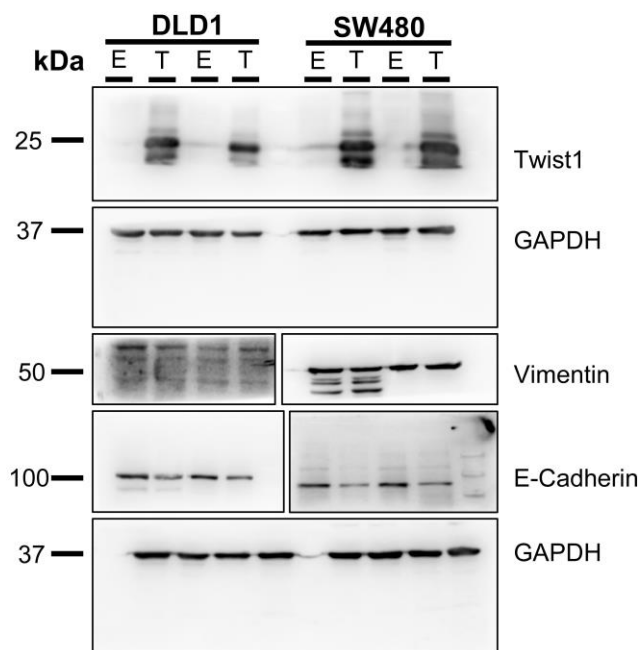

**Fig.2I**

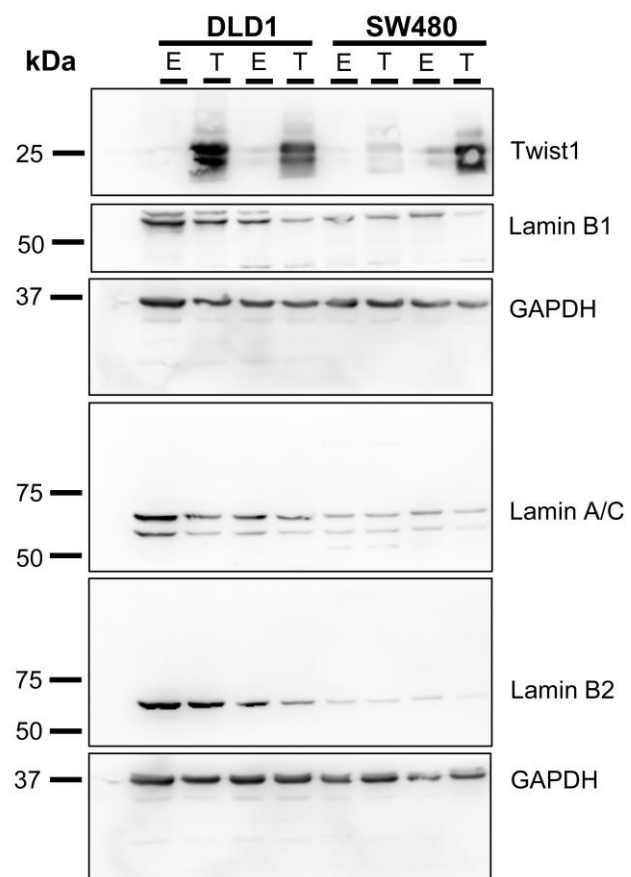



**Fig.4D**

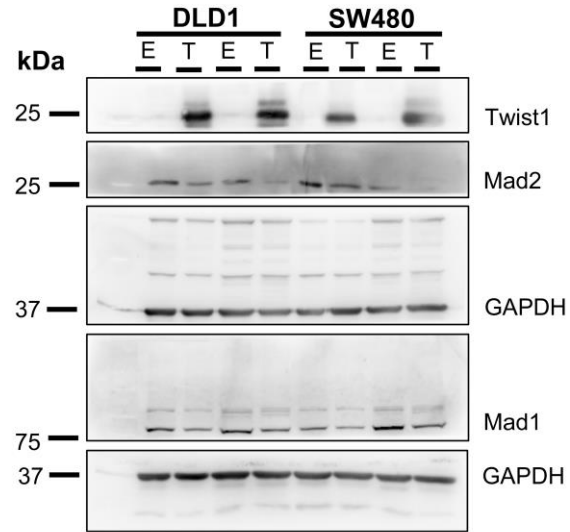

**Fig.4G**

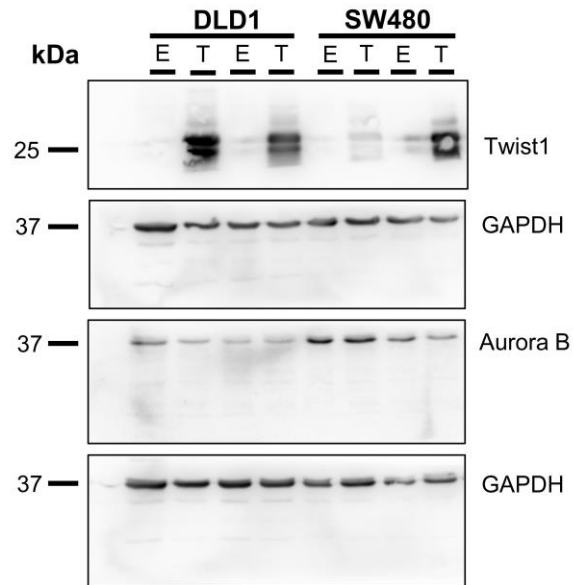

**Fig.4G**

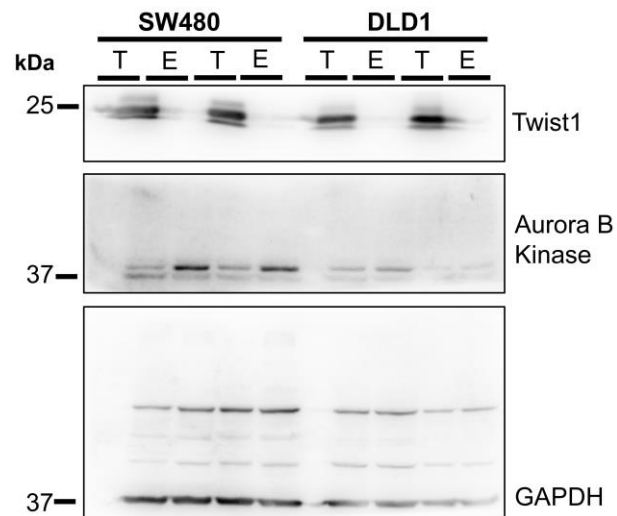

Supplement: Supplementary_Uncropped_blots_ddaa076 [file supplementary_uncropped_blots_ddaa076.pdf]
